# A New Mechanism of the Selective Photodegradation of Antibiotics in the Catalytic System Containing TiO_2_ and the Inorganic Cations

**DOI:** 10.3390/ijms22168696

**Published:** 2021-08-13

**Authors:** Wojciech Baran, Mateusz Cholewiński, Andrzej Sobczak, Ewa Adamek

**Affiliations:** Department of General and Analytical Chemistry, Faculty of Pharmaceutical Sciences in Sosnowiec, Medical University of Silesia in Katowice, Jagiellońska 4, 41-200 Sosnowiec, Poland; wbaran@sum.edu.pl (W.B.); mateochol@tlen.pl (M.C.); asobczak@sum.edu.pl (A.S.)

**Keywords:** molecular mechanism, photocatalysis, sulfonamides, free radicals, TiO_2_

## Abstract

The mechanism of sulfisoxazole (SFF) selective removal by photocatalysis in the presence of titanium (IV) oxide (TiO_2_) and iron (III) chloride (FeCl_3_) was explained and the kinetics and degradation pathways of SFF and other antibiotics were compared. The effects of selected inorganic ions, oxygen conditions, pH, sorption processes and formation of coordination compounds on the photocatalytic process in the presence of TiO_2_ were also determined. The Fe^3+^ compounds added to the irradiated sulfonamide (SN) solution underwent surface sorption on TiO_2_ particles and act as acceptors of excited electrons. Most likely, the SFF degradation is also intensified by organic radicals or cation organic radicals. These radicals can be initially generated by reaction with electron holes, hydroxyl radicals and as a result of electron transfer mediated by iron ions and then participate in propagation processes. The high sensitivity of SFF to decomposition caused by organic radicals is associated with the steric effect and the high bond polarity of the amide substituent.

## 1. Introduction

Demand for food of animal origin results in an increase in the number of pharmaceuticals used in industrial animal husbandry. As a result, approximately 2/3 of currently produced antibiotics are used in veterinary medicine [1,2,3,4,5]. A significant portion of these drugs enters the environment and can pose a serious risk to biocenosis. Sulfonamides (SNs) cause disorders in the metabolism of soil microorganisms at concentrations 10^6^ times lower than their concentration in manure [6]. Antibiotics, even at sub-inhibitory concentrations, promote the generation of drug resistance in microorganisms in the environment [5,6,7,8,9]. Therefore, these pharmaceuticals can also pose a serious problem to human health [6,10]. Additionally, commonly used biological wastewater treatment methods generate a risk of the transfer of antibiotic resistance genes from active sludge to human pathogens [11].

Researchers have high hopes for the use of photocatalytic degradation processes in the presence of TiO_2_ to remove xenobiotics from wastewater. The photocatalytic process allows the effective degradation of antibiotics into biologically inactive products and, simultaneously, decomposition of the genetic material present in manure [12,13,14]. Nowadays, most studies of the photocatalytic degradation of antibiotics have focused on increasing photocatalyst activity and on the use of sunlight [12,15]. Despite the investment of considerable financial resources, however, the problems associated with the application of photocatalytic degradation in wastewater treatment technology have not yet been resolved. The low economic efficiency of the process is unacceptable to food producers. The feasibility of the process is also limited by the level of investment required, the associated operating costs, and its efficiency. Unfortunately, the efficiency of xenobiotics photodegradation in wastewater using simple, low-cost methods is low. One of the reasons for this is the non-selectivity of the photocatalytic process. Much of the energy is consumed in reactions with other, nontoxic wastewater components that are present in large amounts [16]. A considerable improvement in efficiency would be possible if selective degradation of the selected hazardous component of wastewater could be performed.

During UV irradiation of TiO_2_ particles, high-energy pairs, an excited electron (e^−^*) and a hole (h^+^) are generated [12,13]:

TiO_2_ + *hv* → TiO_2_(h^+^; e^−^*) (1)

In an aqueous environment saturated with oxygen, the photoinduced pairs initiate the formation of free radicals, e.g., highly active but unstable hydroxyl (HO^•^), more stable but less active superoxide (O_2_^•−^), hydroperoxide (HO_2_^•^) and organic (R^•^) radicals [12,13,17]:

H_2_O + h^+^ → HO^•^ + H^+^ (2)

O_2_ + e^−^*→ O_2_^•−^ (3)

O_2_ + e^−^* + H^+^ → HO_2_^•^ (4)

RH + h^+^ → R^•^ + H^+^ (5)

Free radicals are very active but react nonselectively. These radicals can react with organic and inorganic compounds or terminate reactions (Equations (6)–(14)):

HO^•^ + RH → RHOH^•^ (6)

HO^•^ + RH → RH^•+^ + OH^−^ (7)

HO^•^ + R^•^ → ROH (8)

HO^•^ + HO^•^ → H_2_O_2_ (9)

O_2_^•−^ + R^•^ → ROO^−^ (10)

HO_2_^•^+ R^•^ → ROOH (11)

R^•^ + O_2_ → ROO^•^ (12)

R^•^ + R_I_H → R−R_I_ → degradation products (13)

R^•^ + R^•^ → R_2_ (14)

Moreover, the organic substances adsorbed on the photocatalyst surface can decompose due to direct charge transfer in a manner that does not depend on the presence of free radicals (Equations (15) and (16)):

RH_(ads)_ + h^+^ → R^+^_ox_ → degradation products (15)

RH_(ads)_ + e^−^*→ R^−^_red_ → degradation products (16)

One of the simple and inexpensive methods that can be used to increase the photocatalytic activity of TiO_2_ is the addition of Fe^3+^ salts to the reaction environment. Promising results after the simultaneous use of TiO_2_ and FeCl_3_ solution were reported by Zhang et al. [18]. This system can intensify the generation of free radicals due to the occurrence of parallel reactions (2) and (17):

Fe(OH)^2+^ + *hv* → Fe^2+^ + HO^•^ (17)

An increase in the photodegradation efficiency of the photocatalytic process performed in the presence of Fe^3+^ salts may also be due to an increase in the sorption of anionic reactants on the TiO_2_ surface, the formation of coordinate bonds with Fe^3+^ ions and the binding of e^−^*. As a result, recombination of the electron-hole pairs is limited (Equation (18)) [18,19,20]:

Fe^3+^ + e^−^*→ Fe^2+^ (18)

According to our previous study, the photodegradation rate of SFF in the presence of TiO_2_/FeCl_3_ was even 100 times higher than that of other SN antibiotics [20]. However, the presented hypotheses did not clearly explain why SFF showed high susceptibility to photocatalytic degradation.

The purpose of our paper is to explain the mechanism of selective photodegradation of SFF in wastewater and in combination with its molecular structure. The primary novelty of this research is a new mechanism of the photocatalytic degradation of SFF with the participation of cation radicals and organic radicals, not only hydroxyl radicals. The proposed mechanism is particularly valuable because it is not found in the literature. In the experiments, we compared not only the photodegradation process between various sulfonamides but also between other pharmaceuticals, including antibiotics and anticancer drugs.

In our opinion, in veterinary settings, the use of selected antibiotics that are susceptible to selective photocatalytic degradation in the presence of simple (inexpensive) catalytic systems may be more appropriate than the use of antibiotics whose degradation requires complex (expensive) high-activity systems. This solution can reduce the problem of the low economic efficiency of the photocatalytic process. However, before introducing these antibiotics in veterinary practice, it is necessary to determine the mechanism of their selective susceptibility.

## 2. Results and Discussion

### 2.1. Photocatalytic Degradation of Drugs in Wastewater—Screening Tests

**Figure 1:**
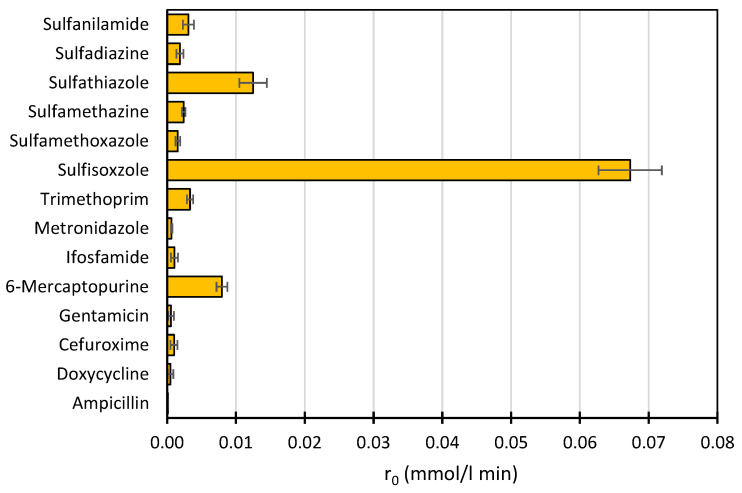
Comparison of the initial photodegradation rates of selected pharmaceuticals in synthetic wastewater in the presence of TiO_2_ (0.5 g/L)/FeCl_3_ (1.0 mmol/L).

Previously published results on SNs photocatalytic degradation [16,20] prompted us to perform screening tests on the susceptibility of various pharmaceuticals (Table S1, Supplementary Materials) to photocatalytic degradation in synthetic wastewater (Table S2, Supplementary Materials) in the presence of a TiO_2_ (0.5 g/L)/FeCl_3_ (1.0 mmol/L) mixture. All reactions were conducted at pH 3.0 ± 0.1 (Section 3.2). The results are shown in Figure 1.

Under the experimental conditions used, the photodegradation rate of SFF in wastewater was considerably higher than that of the other drugs. At the same time, in contrast to the other SNs, the degradation rate of SFF in a complex matrix decreased the least compared to the rate in distilled water [20]. This indicates that the studied photocatalytic process can be used for the efficient and selective removal of SFF from wastewater. We performed the experiments described in this paper to determine this.

### 2.2. Identification of Photocatalytic Degradation Products of the Selected SNs

Aqueous solutions (0.1 mmol/L) of SFF, sulfathiazole (STZ) and sulfanilamide (SAD) were used in the experiments in which we identified photodegradation products. The experiments were performed in the presence of a mixture of TiO_2_ (0.5 g/L)/FeCl_3_ (1.0 mmol/L) at pH 3.0 ± 0.1. Additionally, products of SFF (0.1 mmol/L) photodegradation conducted in the presence of TiO_2_ alone (0.5 g/L) at pH in the range of 4.4–7.5 and in the presence of FeCl_3_ alone (1.0 mmol/L) at pH 3.0 ± 0.1 were identified using MSMS technique (Section 3.8). The results are summarized in Table 1. Appropriate chromatograms and MS/MS spectra are included in the Supplementary Materials (Figure S1).

Among the photodegradation products of SFF that were formed in the presence of TiO_2_/FeCl_3,_ only two of nine identified products, i.e., C_8_H_13_NO and C_9_H_14_N_2_O_3_, possessed –OH group(s). These compounds were absent from solutions that had been irradiated for less than 30 s (Figure S1a, Supplementary Materials) and were likely products of subsequent stages of SFF degradation. In turn, a few compounds containing –OH group(s) attached to the aromatic (C_12_H_12_N_4_O_5_S_2_) or heterocyclic (C_9_H_11_N_3_O_4_S_2_) ring were identified among the initial products of SAD and STZ photodegradation (Table 1). This may indicate that these SNs reacted with HO^•^ radical (in contrast to SFF) [21].

During the photodegradation of all studied SNs in the presence of TiO_2_/FeCl_3_, many products with molecular masses significantly higher than the masses of the parent compounds were formed. These compounds likely resulted from the condensation of SNs and/or their degradation products and might possess azo bonds.

A condensation product of two C_11_H_14_N_3_O_4_S molecules (C_22_H_22_N_6_O_8_S_2_; Table 1) was also identified after SFF photodegradation in the presence of FeCl_3_. Among six identified compounds, four possessed −OH group(s). SAD (C_6_H_8_N_2_O_2_S) and 1-amino-3,4-dimethylisoxazole (C_5_H_8_N_2_O) were also identified.

An unusual, extended peak with a retention time of 2.10 min and m/z 199.1078 Da (Figure S1a,b) was observed on the chromatograms after photodegradation of SFF in the presence of a TiO_2_/FeCl_3_ mixture and after photodegradation of SFF in the presence of FeCl_3_ alone. Based on QTof analysis, the compound in this peak may have the formula C_9_H_14_N_2_O_3_. We suppose that this compound formed as a result of the decomposition of iron complex(es) in the ion source of the mass spectrometer.

After SFF photodegradation in the presence of TiO_2_ alone at pH 4.4–7.5, most of the identified products had –OH group(s) attached directly to the original SN structure or to its nitrosyl derivative. An SFF structure with an –OH group attached to the benzene ring (C_11_H_14_N_3_O_4_S, Table 1) was identified in the first sample taken after 1 min of irradiation. In contrast to reactions performed in the presence of TiO_2_/FeCl_3_ and FeCl_3_, condensation products of SFF were not identified when the reactions were performed in the presence of TiO_2_ at pH 4.4–7.5 (Table 1). However, these compounds were observed during the photocatalytic degradation of SNs with TiO_2_ at lower pH (results not shown). The formation of these condensation compounds is also possible during the Fenton process [17].

The abovementioned results confirm that the mechanisms of SNs photodegradation conducted in the presence of various catalytic systems differed. We suspect that when TiO_2_ or FeCl_3_ was used as the catalyst, the main pathway of SFF photodegradation proceeded through the HO^•^ radical. This radical can also initiate the degradation of SAD and STZ in the presence of a TiO_2_/FeCl_3_ mixture. No evidence of the involvement of HO^•^ radicals in the initiation of SFF degradation in this catalyst system was found.

According to Ge et al. [21], degradation of five-membered SNs can proceed as a result of direct photolysis without the participation of HO^•^ radicals. SFF is more susceptible than other SNs to photolysis initiated by UVC radiation [22]. However, under direct photolysis, the degradation products of SFF should be primarily sulfanilic acid and 5-amino-3,4-dimethylisoxazole [21]. In our experiments, those compounds were not detected in the solution after the photocatalytic degradation of SFF with a TiO_2_/FeCl_3_ mixture. Moreover, SFF does not differ from other SNs in its rate of photodegradation initiated by UVA radiation with TiO_2_ [20]. The presence of condensation products in solutions after photocatalysis suggests that the transformation of SNs may be initiated not through HO^•^ radicals but through organic radicals or cation radicals (Equations (13) and (14)). These radicals, which form according to Equations (5)–(7), can diffuse into the bulk solution and react with SNs that are not adsorbed on the catalyst surface. Radicals formed as a result of irradiation of coordination compounds of Fe^3+^ (Equations (19) and (20)) may also affect the intensification of the discussed process [17,23].

(19) $$\mathrm{Fe}(\mathrm{OH})(\mathrm{RH}{)}^{2+}\;\overset{hv\left(pH\;3\right)}{\to }\;{\mathrm{Fe}(\mathrm{OH})}^{+}+R^{•}+H^{+}$$

(20) $$\mathrm{Fe}(\mathrm{OH})(R{)}^{2+}\;\overset{hv\left(pH\;3\right)}{\to }\;{\mathrm{Fe}(\mathrm{OH})}^{+}+R^{•+}$$

The condensation products can also form as a result of charge transfer reactions (Equation (21)).

(21) $$\mathrm{Fe}(\mathrm{OH})(\mathrm{RH}{{)}_{2}}^{2+}\;\overset{hv\left(pH\;3\right)}{\to }\;\mathrm{Fe}(\mathrm{OH}{)}^{+}+R-\mathrm{RH}+H^{+}$$

Epoxides, which indicate the peroxides formed during the reaction, were not identified among the products analyzed [24].

SFF degradation products, e.g., SAD and its dimers and dimethylisoxazole adducts, exhibit a breakdown of the bond between the amide nitrogen and the heterocyclic substituent. This was reported in 1973 by Manzo et al. [25]. In turn, 1-amino-3,4-dimethylisoxazole, which formed in the presence of FeCl_3_, could be the product of hydrolysis of the amide group in SFF. In this case, sulfanilic acid should also form. This compound is often found among the products of photocatalytic degradation of other SNs [26,27,28]. However, sulfanilic acid was not identified in the solutions after SFF degradation.

Based on the products we identified, we suspect that HO^•^ radicals do not participate in the main pathway of SFF photodegradation in the presence of TiO_2_/FeCl_3_. Most likely, due to the acidic reaction environment, propagation reactions involving organic radicals and/or cation radicals are of key importance for SFF photodegradation. A parallel, light-initiated charge transfer within the SFF-iron complex is also possible.

### 2.3. Study of the Kinetics of Photocatalytic Degradation of SFF in the Presence of a TiO_2_/FeCl_3_ Mixture

Preliminary studies have confirmed that the SFF degradation reaction that occurs in the presence of TiO_2_/FeCl_3_ proceeds according to pseudo-first-order kinetics and that its initiating factor is UVA radiation. In the dark, SFF did not undergo degradation after adsorption equilibrium was established (Figure S2, Supplementary Materials).

#### 2.3.1. pH Effect

**Figure 2:**
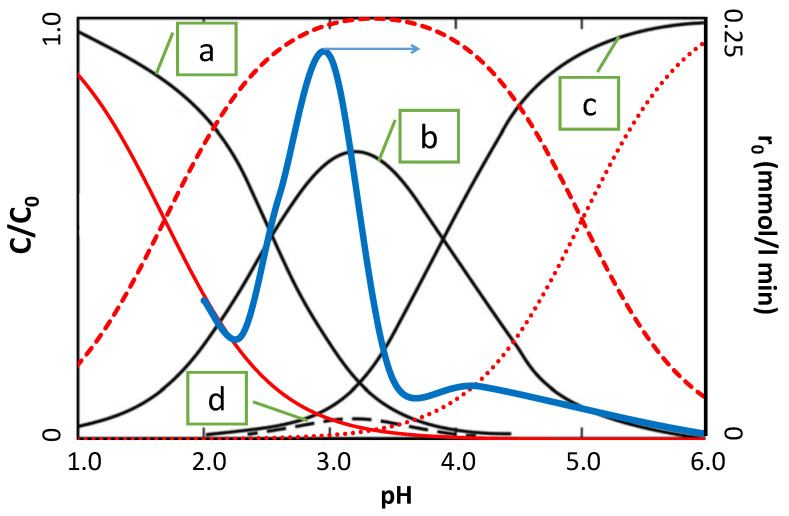
Effect of the pH of the aqueous solution on various parameters of the photocatalytic transformation of SFF (0.1 mmol/L) in the presence of a mixture of TiO_2_ (0.5 g/L) and FeCl_3_ (1.0 mmol/L): rate of photodegradation of SFF (blue line); concentration of the cationic form of SFF (solid red line); concentration of the neutral form of SFF (red dashed line); concentration of the anionic form of SFF (red dotted line); (**a**) concentration of [Fe(H_2_O)_6_]^3+^ ions; (**b**) concentration of [Fe(H_2_O)_5_(OH)]^2+^ ions; (**c**) concentration of [Fe(H_2_O)_4_(OH)_2_]^+^ ions; (**d**) concentration of [Fe_2_(H_2_O)_8_(OH)_2_]^4+^ ions.

The pH of the irradiated solution has a strong effect on the kinetics of photocatalytic degradation of SNs in the presence of TiO_2_/FeCl_3_ [29]. The relationship between the photocatalytic degradation rate of SFF (0.1 mmol/L) and pH in the range 1 to 6 is shown in Figure 2. The process was conducted in the presence of a TiO_2_ suspension (0.5 g/L) and FeCl_3_ solution (1.0 mmol/L). Detailed information on the results obtained over a wider pH range is provided in Figure S3 (Supplementary Materials).

The optimal pH for SFF photodegradation in the presence of TiO_2_/FeCl_3_ was observed to fall within a narrow range around pH~3. Increasing or decreasing the pH resulted in strong inhibition of the reaction. Analogous results were described for other reactants degraded in the presence of TiO_2_/Fe^3+^ [18,20,30,31,32,33,34]. According to the majority of researchers, this effect is likely related to the presence of photochemically active Fe(OH)^2+^ ions in the Fe^3+^—salt solution at pH~3. The significant inhibition of the reaction at higher pH can be explained by the further hydrolysis of iron compounds to inactive Fe(OH)^2+^ ions, followed by the formation of Fe(OH)_3_ and Fe(OH)_4_^−^ [35]. These compounds could block access of SFF to the TiO_2_ surface and hinder the formation of complex compounds that may participate in the photocatalytic process.

The addition of Fe^3+^ salt to the photocatalyst suspension can also affect the conditions under which reactant sorption occurs. At pH~3, the molar fraction (X) of the molecular form of SFF was higher than that of the other SNs studied (Table S3, Supplementary Materials). In our opinion, the optimal conditions that favor the cumulation of neutral SFF molecules at a positively charged surface of the TiO_2_/FeCl_3_ system occur at this pH. Under these conditions, the anionic forms of SNs should be adsorbed to a much greater extent; therefore, the photodegradation rates of SAD (X_S−_ = 0.224) and STZ (X_S−_ = 0.142) should be much higher than that of SFF (X_S−_ = 0.056). In fact, the photodegradation rate of SFF was the highest. This suggests that the anionic forms of SNs are less susceptible to photodegradation than neutral particles. This conclusion may be of key importance when considering the reaction rate.

#### 2.3.2. Effect of the Initial Concentration of SFF

**Figure 3:**
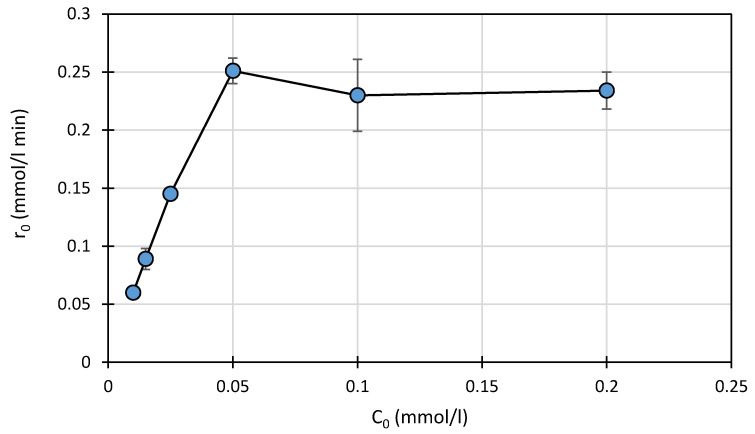
Effect of the initial SFF concentration on the photodegradation rate in the presence of a mixture of TiO_2_ (0.5 g/L) and FeCl_3_ (1.0 mmol/L) at pH 3.0 ± 0.1.

The relationship between the initial concentration of SFF (0.01–0.20 mmol/L) and its photodegradation rate is shown in Figure 3. The reaction was performed at pH 3.0 ± 0.1 in the presence of a TiO_2_ (0.5 g/L)/FeCl_3_ (1.0 mmol/L) mixture.

Increasing the initial SFF concentration from 0.01 to 0.05 mmol/L resulted in a linear increase in the reaction rate (Figure 3). However, increasing the SFF concentration beyond 0.05 mmol/L did not increase the initial degradation rate, and the reactant concentration hardly affected the r_0_ value.

The relationship between the degree of adsorption and the initial SFF concentration showed a similar pattern (Figure S4, Supplementary Materials). An increase in adsorption from ~1.5 to ~5% was observed only when the initial SFF concentration was ≤0.05 mmol/L. The reactant molecules most likely occupied the available active sites on the catalyst surface until saturation occurred at a concentration of 0.05 mmol/L. This indicates that, according to the Langmuir–Hinshelwood theory (L–H), the reaction rate is limited by the number of active sites on the photocatalyst [36,37,38].

**Figure 4:**
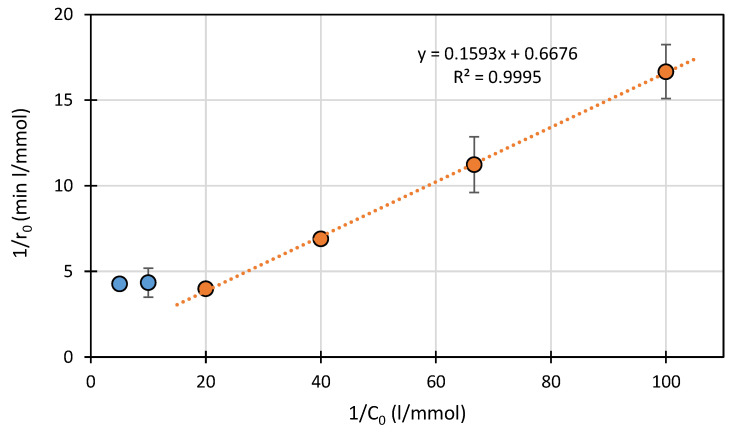
Plot of 1/r_0_ as a function of 1/C_0_ for SFF photodegradation in the presence of TiO_2_/FeCl_3_.

This assumption is confirmed by the linearity of the relationship 1/r_0_ = f(1/C_0_) for SFF at concentrations <0.05 mmol/L (Figure 4). Based on these results, we assume that sorption processes can be of significant importance and may be a factor that limits the photodegradation rate of SFF in the presence of TiO_2_/FeCl_3_.

#### 2.3.3. Effect of TiO_2_ Amount

**Figure 5:**
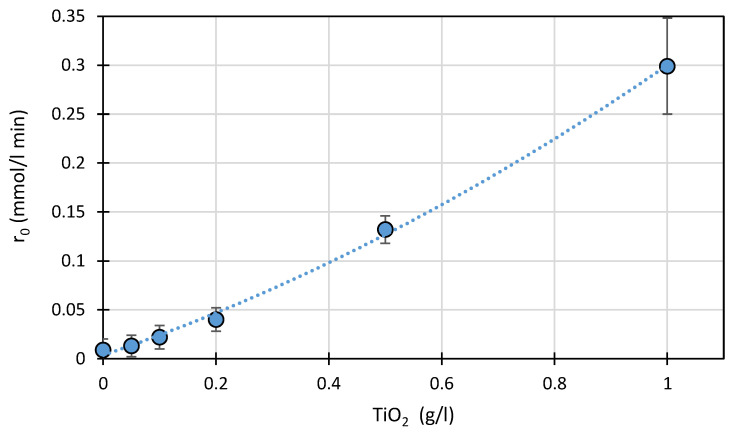
Effect of the amount of TiO_2_ present on the photodegradation rate of SFF (0.1 mmol/L) in the presence of FeCl_3_ (1.0 mmol/L) at pH 3.0 ± 0.1.

The effect of TiO_2_ amount (0–1 g/L) on the initial rate of photocatalytic degradation of SFF (0.1 mmol/L) in the presence of FeCl_3_ (1.0 mmol/L) at pH 3.0 ± 0.1 is shown in Figure 5.

Under these conditions, increasing the concentration of TiO_2_ resulted in a monotonic increase in the rate of SFF photodegradation. This result is consistent with the L–H theory because the presence of a larger number of catalyst particles is associated with a greater number of active sites. On the other hand, a 20-fold increase in the amount of TiO_2_ present (at a constant FeCl_3_ concentration) resulted in only a slight increase in the SFF adsorption rate from ~4 to ~6% (Figure S5, Supplementary Materials).

According to many studies, the initial reaction rate increases as the photocatalyst amount increases up to a certain limit; beyond that limit, it does not accelerate or even slows down [39,40,41]. This could be due to excessive light scattering by the suspended TiO_2_ particles. However, we found no such correlation in our experiments.

**Figure 6:**
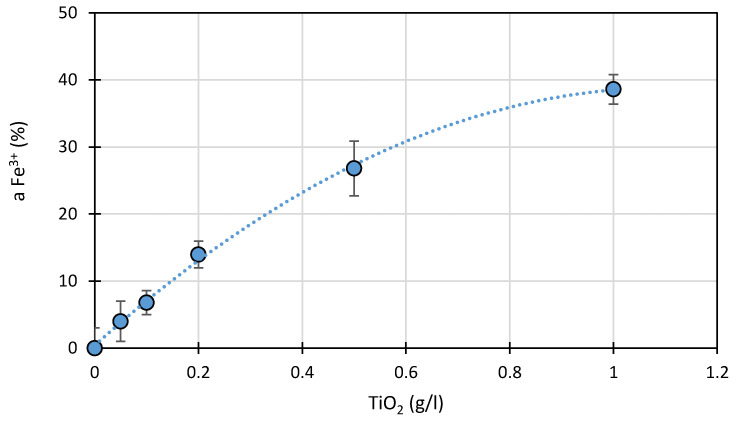
Effect of the amount of TiO_2_ present on the sorption (a) of Fe^3+^ compounds from FeCl_3_ solution (1.0 mmol/L) at pH 3.0 ± 0.1.

The amount of TiO_2_ present has a significant effect on the binding of FeCl_3_ hydrolysis products (Figure 6). In both the anatase and rutile crystals, the titanium ion (Ti^4+^) is surrounded by six oxygen ions (O^2−^) [42]. Therefore, the formation of coordination bonds between oxygen anions from TiO_2_ and Fe^3+^ is possible. It is very likely that adsorption of Fe^3+^ compounds on the TiO_2_ surface may be important in enhancing photocatalytic processes [18,30].

#### 2.3.4. Effect of FeCl_3_ Concentration

**Figure 7:**
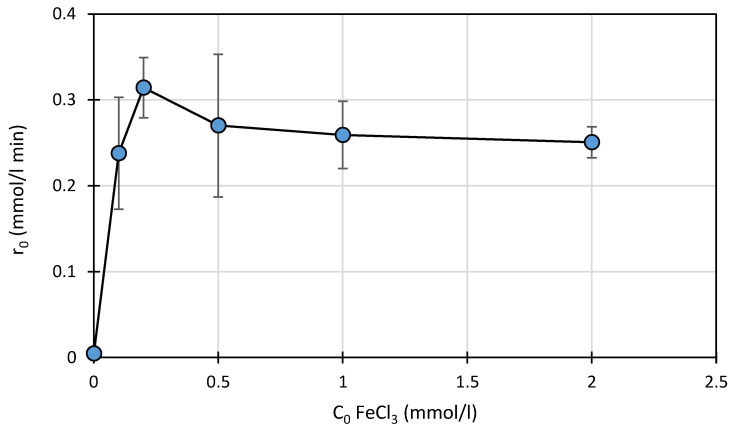
Effect of the initial FeCl_3_ concentration on the photodegradation rate of SFF (0.1 mmol/L) in the presence of TiO_2_ (0.5 g/L) at pH 3.0 ± 0.1.

The relationship between the FeCl_3_ concentration (0–2.0 mmol/L) and the rate of photodegradation of SFF (0.1 mmol/L) in the presence of TiO_2_ (0.5 g/L) is shown in Figure 7.

SFF exhibited the highest photodegradation rate at a FeCl_3_ concentration of 0.2 mmol/L. A further increase in the FeCl_3_ concentration to 2.0 mmol/L resulted in a slight decrease in the photodegradation rate. It is possible that a higher Fe-salt concentration reduces the radiant flux that reaches the surface of TiO_2_ particles. In addition, polymeric products of hydrolysis (e.g., Fe_2_(OH)_2_^4+^) could form at higher FeCl_3_ concentrations at pH 3 [35]. These compounds do not have photocatalytic properties and may block access to active sites in the catalytic system. It cannot also be excluded that FeCl_3_ concentration equal to 0.2 mmol/L is optimal from the viewpoint of SFF sorption and desorption of reaction products.

According to certain studies, Cl^−^ ions inhibit the photocatalytic process due to their reaction with highly reactive HO^•^ radicals (Equation (22)). Therefore, the supply of HO^•^ radicals in other reactions can be reduced [43,44,45].

HO^•^ + Cl^−^ → HO^−^ + Cl^•^ (22)

In our opinion, due to their high potential and longer lifetime (Table S4, Supplementary Materials), Cl^•^ radicals may cause an increase in the degradation rates of reactants that are not adsorbed on the TiO_2_ surface. However, after SFF photodegradation with TiO_2_/FeCl_3_, none of the analyzed samples exhibited the presence of chlorine-containing products (Table 1) (theoretically, these products could form under the conditions used here) [45].

#### 2.3.5. Formation of Fe^3+^ Complexes with SNs

The formation of thermodynamically stable coordination compounds of Fe^3+^ with SNs was described previously [17,46,47]. In these compounds, SN can serve as a chelating ligand.

**Figure 8:**
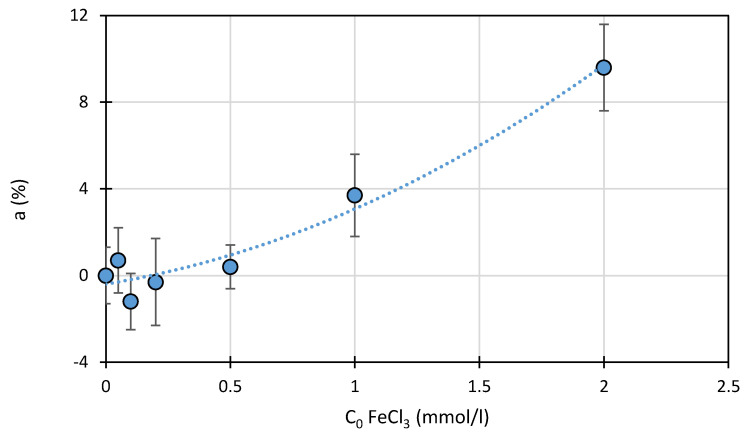
Effect of the initial FeCl_3_ concentration on the binding (a) of SFF (1.0 mmol/L) in the dark at pH 3.0 ± 0.1.

Figure 8 presents the relationship between the FeCl_3_ concentration (0.05–2.0 mmol/L) and the amount of bound SFF (0.1 mmol/L) in solutions that had been mixed for 30 min in the dark at pH 3.0 ± 0.1 (Section 3.7). A slight monotonic increase in SFF binding was observed in solutions containing FeCl_3_ at a concentration of >0.5 mmol/L. The degree of SFF binding was approximately 10% even when Fe salt was present in 20-fold excess.

The degree of SFF binding to Fe^3+^ that was significantly lower than expected could be caused by the fact that the resulting [Fe^3+^(SFF)]^n+^ complex was soluble (not removed by centrifugation) and, simultaneously, unstable under UPLC analysis.

### 2.4. Comparison of the Photocatalytic Degradation of SFF with That of Other SNs

**Figure 9:**
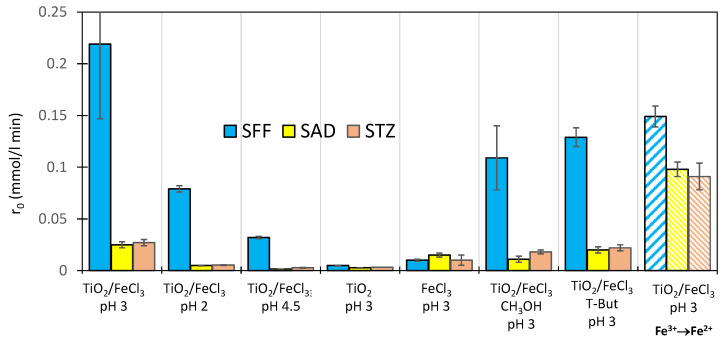
Comparison of the initial rates of photocatalytic degradation of SFF, SAD and STZ (0.1 mmol/L) with the rate of Fe^3+^ reduction in the presence of TiO_2_ (0.5 g/L) and/or FeCl_3_ (1.0 mmol/L).

To determine the reasons for the higher susceptibility of SFF to photodegradation, the effect of basic parameters of the photocatalytic process on SAD and STZ degradation was studied (Section 3.4). The results are shown in Figure 9.

With the exception of the reaction performed in the presence of FeCl_3_ alone, SFF photodegradation occurred at a significantly higher rate than SAD and STZ photodegradation. In the presence of TiO_2_/FeCl_3_ at pH 3, the photodegradation rate of SFF was over eight times higher than that of SAD and STZ. In the presence of TiO_2_ alone, the SFF photodegradation rate was two times higher than that of the other SNs, and in the presence of FeCl_3_ alone, the photodegradation rate of SAD was the highest. The degradation rates of SFF, SAD and STZ in the TiO_2_/FeCl_3_ mixture were ~15, 1.4 and 2 times higher, respectively than the algebraic sums of the reaction rates determined separately for the processes conducted with TiO_2_ and FeCl_3_. It is excluded that the high degradation rate of SFF results from a simple, synergistic generation of HO^•^ radicals in parallel reactions (Equations (2) and (17)). On the other hand, this synergistic effect may also be important in determining the photodegradation rates of SAD and STZ (Figure 9).

### 2.5. Total Organic Carbon (TOC) Removal

**Figure 10:**
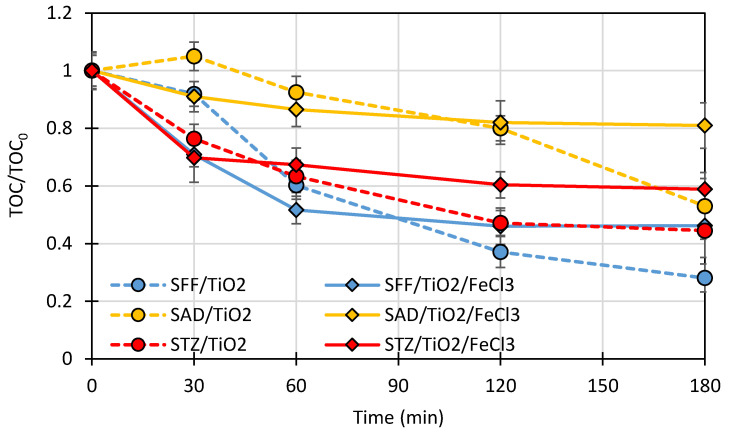
TOC removal during the photocatalytic degradation of SFF, SAD and STZ (0.1 mmol/L) in the presence of TiO_2_ (0.5 g/L) and TiO_2_ (0.5 g/L)/FeCl_3_ (1.0 mmol/L).

The dynamics of TOC removal expressed as TOC/TOC_0_ are shown in Figure 10.

TOC removal is a measure of the mineralization of organic compounds in the sample (Section 3.9). The total photocatalytic mineralization of compounds can be much slower than their photocatalytic degradation. During 180 min experiments, only partial mineralization of the studied SNs was achieved. The addition of FeCl_3_ to the SN/TiO_2_ mixture had a negative effect on the process efficiency.

The aim of the photocatalytic removal of antibiotics from the aquatic environment is primarily to remove their antimicrobial activity. It was found that even partial photocatalytic mineralization of antibiotic solutions resulted in the disappearance of their antimicrobial activity [16]. Therefore, a longer UV-irradiation leading to full mineralization of SNS is economically unjustified.

### 2.6. Kinetics of Fe^3+^ Reduction during SNs Photodegradation in the Presence of a Mixture of TiO_2_/FeCl_3_

As mentioned above, during irradiation of solutions containing photoactive Fe^3+^-compounds, e.g., Fe(OH)^2+^, Fe^3+^ ions can be reduced to Fe^2+^ ions (Equation (17)). Previous results confirm that the Fe^2+^ concentration increases in a manner that is inversely proportional to the Fe^3+^ concentration during UVA irradiation of the TiO_2_/FeCl_3_ mixture [29]. Therefore, an increased supply of HO^•^ radicals should result from this reduction. Additionally, it cannot be excluded that Fe^3+^ ions can also act as acceptors of photoexcited electrons on the TiO_2_ surface (Equation (18)) [30].

Then, the prevention of recombination between e^−^* and h^+^ occurs, and as a consequence, the supply of h^+^ in the chain of reactions initiating SN degradation increases. It is possible that both mechanisms have a significant effect on the photodegradation rate of STZ and SAD.

Fe^2+^ ions can be reoxidized to Fe^3+^ using oxidants formed in the irradiated solution (Equations (23) and (24)):

Fe^2+^ + h^+^ → Fe^3+^ (23)

Fe^2+^ + HO^•^ → Fe^3+^ + HO^−^ (24)

Each of these processes decreases the overall rate of Fe^3+^ reduction but, simultaneously, can also inhibit reactions with h^+^ and HO^•^.

The rate of Fe^3+^ reduction depended on the type of SN (Figure 9, dashed bars, and Figure S6, Supplementary Materials). The highest Fe^3+^ reduction rate was observed in samples containing SFF; however, the parallel photodegradation of this drug occurred at a significantly higher rate. In contrast, in the SAD and STZ samples, the rate of reduction of Fe^3+^ was approximately ~3.5–4 times higher than the photodegradation rate (Figure 9).

In our opinion, during photodegradation in the presence of TiO_2_/FeCl_3_, Fe^3+^ ions likely mediate the transfer of electrons from complexed SNs to h^+^. In the transient stage, a complex with Fe^4+^ may be formed [17,48,49], and Fe^3+^, Fe^2+^ ions and oxidized SN may be the product of its reduction (Equation (25)):

(25) $$[{\mathrm{TiO}}_{2}-{\mathrm{Fe}}^{3+}(\mathrm{SN})]\;\overset{hv}{\to }\;[{\mathrm{TiO}}_{2}-{\mathrm{Fe}}^{4+}(\mathrm{SN})]\;\to \;{\mathrm{TiO}}_{2}-{\mathrm{Fe}}^{3+}+{\mathrm{Fe}}^{2+}+{\mathrm{SN}}_{\mathrm{ox}}$$

This mechanism does not include the participation of HO^•^ radicals in the initiation of SN degradation. Additionally, if the [TiO_2_-Fe^3+^(SFF)] complex differs significantly in stability or formation rate from the TiO_2_-Fe^3+^ complex formed with other SNs these complexes will significantly differ in the photocatalytic degradation rate. Undoubtedly, the processes leading to the reduction of Fe^3+^ ions (e.g., Equations (17) and (18)) have a positive effect on the rate of SNs photodegradation in the TiO_2_/FeCl_3_ mixture.

### 2.7. Fenton-Like Reactions

It is possible that, simultaneously with the photocatalytic process and in the presence of Fe^3+^ salts, Fenton-like reactions proceeded with the participation of formed H_2_O_2_ molecules (Equations (9), (26) and (27)) [17,18,19,20,21,22,23,24,25,26,27,28,29,30,31,32,33,34,35,36,37,38,39,40,41,42,43,44,45,46,47,48,49,50,51]:

HO_2_^•^ + RH → R^•^ + H_2_O_2_ (26)

Fe^3+^ + H_2_O_2_ + H^+^ → Fe^2+^ + HO_2_^•^ + H_2_O (27)

Fe^2+^ ions (Equation (27)) formed in this process can also participate in the generation of HO^•^ radicals (Equation (28)):

Fe^2+^ + H_2_O_2_ + H^+^→ Fe^3+^ HO^•^ + H_2_O (28)

In this case, the radicals appear in the bulk solution and not on the catalyst surface. They can potentially react with reactant that is not adsorbed on the TiO_2_ surface. However, in the Fenton process, SFF does not differ from other SNs in its susceptibility to degradation (Figure S7, Supplementary Materials); therefore, that mechanism is unlikely.

### 2.8. Photodegradation of SNs in the Presence of a TiO_2_/FeCl_3_ Mixture with the Addition of Methanol or Tert-Butanol

The addition of excess (100×) methanol and tert-butanol (t-But) has often been used to determine the mechanisms of chemical processes [28,38,44,50]. These compounds, as scavengers, are added to the solutions to inhibit the action of h^+^ or HO^•^ radicals (Equations (29)–(31)):

CH_3_OH + h^+^ → CH_2_OH^•^ + H^+^ (29)

CH_3_OH + HO^•^ → CH_2_OH^•^ + H_2_O (30)

C_4_H_9_OH + HO^•^ → C_4_H_8_OH^•^ + H_2_O (31)

The addition of methanol (10 mmol/L) caused a ~50% decrease in the rate of SFF and SAD photodegradation and a ~30% decrease in the rate of STZ photodegradation. In turn, t-But (10 mmol/L) slowed the photodegradation of these SNs by ~40%, 20% and 20%, respectively (Figure 9). Although the large 100-fold excess of methanol and t-But relative to the SNs, no complete inhibition of the photodegradation of these drugs was found. These results indicate that HO^•^ radicals involved in the initiation of SFF degradation and h^+^ participated in the initiation of SAD degradation. However, it can be concluded that the contribution of these active species was not the most important from the viewpoint of the degradation rate of SNs. In the case of STZ, the supply of h^+^ and HO^•^ has no significant effect on the photodegradation rate. On the other hand, hydroxymethyl (CH_2_OH^•^) and 2-hydroxy-2-methyl-propyl (C_4_H_8_OH^•^) radicals also form in these reactions. The possibility that these radicals participate in propagation reactions with SNs cannot be excluded (Equations (8)–(14)).

### 2.9. Photocatalytic Degradation of SNs in the Presence of a TiO_2_/FeCl_3_ Mixture under Aerobic and Anaerobic Conditions

**Figure 11:**
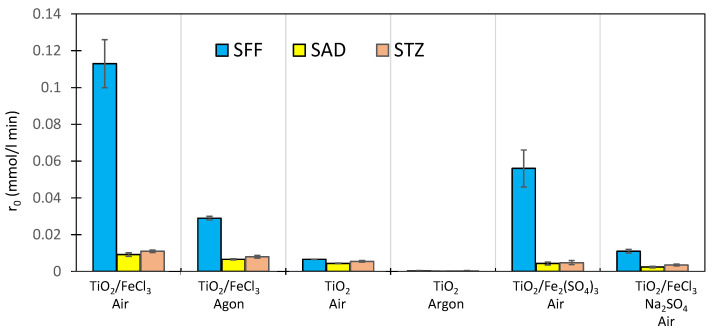
Comparison of the initial rates of photodegradation of SFF, SAD and STZ (0.1 mmol/L) in the presence of TiO_2_ (0.5 g/L) and TiO_2_ (0.5 g/L)/Fe^3+^ salt (1.0 mmol/L) at pH 3 in samples ventilated with air or argon.

Dissolved oxygen is an important reactant in photocatalysis, and it acts mainly as an acceptor of excited electrons (Equations (3), (4) and (12)) [17,42,50]. Additionally, it is a source of HO_2_^•^ radicals in processes conducted in acidic environments (Equation (4)). Removal of oxygen from the reaction environment promotes the recombination of the h^+^/e^−^* pair and consequently inhibits the reactions of SNs with h^+^, HO^•^ and HO_2_^•^. Figure 11 shows the photodegradation rates of the tested SNs (0.1 mmol/L) in the presence of TiO_2_ (0.5 g/L) and a mixture of TiO_2_/Fe^3+^ salt (1.0 mmol/L) in solutions ventilated with air or argon (Section 3.5). In argon-ventilated solutions, the dissolved oxygen concentration was below 0.5% saturation at 295 K. All reactions were performed at pH 3.0 ± 0.1.

Under anaerobic conditions, the photocatalytic degradation of SNs in the presence of TiO_2_ was almost completely inhibited, whereas, in the presence of TiO_2_/FeCl_3,_ it only slowed down. Compared to the reaction with TiO_2_/FeCl_3_/air, the photodegradation rates of SFF, SAD and STZ decreased by ~80%, 28% and 27%, respectively. These results confirm the hypothesis that in the presence of TiO_2,_ Fe^3+^ ions act as acceptors of e^−^* and prevent their recombination with h^+^ [18,19]. In our opinion, this process exerts a decisive influence on the photodegradation rate of SAD and STZ. Moreover, Fe(OH)^2+^ ions adsorbed on the TiO_2_ surface can participate in this process by generating an additional quantity of HO^•^ radicals (Equation (32)):

Fe(OH)^2+^ + e^−^* → Fe^2+^ + HO^•^ (32)

The significant effect of oxygen on the photodegradation rate of SFF in the presence of TiO_2_/FeCl_3_ (Figure 10) indicates that the mechanism of degradation of SFF differs from that of SAD and STZ.

In an acidic environment in the presence of oxygen and Fe salts, (FeO_2_)^2+^ ions containing Fe^(IV)^ may be generated [17,19]. In an aqueous solution, these ions are relatively stable and are active enough that they can even oxidize alkanes [52]. It cannot be excluded that (FeO_2_)^2+^ ions participate in SFF oxidation in the presence of TiO_2_/FeCl_3_.

Lack of oxygen in the reaction environment may also inhibit some propagation reactions of organic radicals (Equation (33)).

(33) $$R^{•}\;\overset{O_{2}}{\to }\;{\mathrm{ROO}}^{•}\;\overset{H_{2}O}{\to }\;\mathrm{ROH}+{{\mathrm{HO}}_{2}}^{•}$$

However, hydroxyl derivatives were formed during this process. As already mentioned, these compounds were not detected in the solution after SFF photodegradation. Therefore, we assume that this process is not a significant determinant of the photodegradation rate of SFF.

### 2.10. Effect of SO_4_^2−^ Ions on the Photocatalytic Degradation of SNs

Photocatalytic degradation of the studied SNs (0.1 mmol/L) in the presence of TiO_2_ (0.5 g/L) and Fe_2_(SO_4_)_3_ (0.5 mol/L) at pH 3.0 ± 0.1 proceeded two times slowly that was observed in the presence of the TiO_2_/FeCl_3_ mixture (Figure 11). However, a stronger inhibitory effect on SNs photodegradation was observed during irradiation of aerated solutions in the presence of TiO_2_/FeCl_3_/Na_2_SO_4_ (3 mmol/L). Photodegradation of SNs in the presence of alone Fe_2_(SO_4_)_3_ proceeded more than three times more slowly than in the presence of alone FeCl_3_ [53]. This indicates that SO_4_^2−^ ions inhibit the photocatalytic process. SO_4_^2−^ ions can react with h^+^ and HO^•^ (Equations (34) and (35)) to form SO_4_^•−^ radicals [45].

SO_4_^2−^ + h^+^ → SO_4_^•−^ (34)

SO_4_^2−^ + HO^•^ → SO_4_^•−^ + OH^−^ (35)

They have a high oxidizing potential (Table S4, Supplementary Materials) and can initiate the decomposition of many organic substances, including SNs [45,54,55,56]. Therefore, we suppose that the abovementioned processes (Equations (37) and (38)) did not slow the degradation of SFF.

The presence of SO_4_^2−^ ions in the samples may change the surface charge of the catalyst suspension particles and impede the sorption of neutral reactant particles. It was confirmed that increasing the Na_2_SO_4_ concentration causes only a slight decrease in the amount of SFF bound by particles in the TiO_2_/FeCl_3_ mixture (Figure S8, Supplementary Materials).

The thermodynamic stability of the [Fe(SO_4_)]^+^ complex is ten times higher than that of the [FeCl]^2+^ complex [57]. In addition, the SO_4_^2−^ ion can associate with Fe^3+^ ions via two coordination bonds. For these reasons, we assume that SO_4_^2−^ ions limit the formation of the [TiO_2_-Fe(SFF)] complex. In the presence of sulfates (VI), SFF photodegradation was inhibited more strongly than was the photodegradation of other SNs; thus, the reaction rate of SFF is more dependent on the formation of the [TiO_2_-Fe(SFF)] complex.

### 2.11. Comparison of the Susceptibilities of the Selected SNs to Photocatalytic Degradation

The particularly high susceptibility of SFF to photodegradation in the presence of TiO_2_/FeCl_3_ may be associated with the high polarity of the bond between the amide nitrogen and the heterocyclic substituent (N_amid_-C) [20]. The electroaffinity of the heterocyclic substituent is correlated with the nature of this bond. This parameter also quantitatively affects the dissociation (Table S3, Figure S9, Supplementary Materials).

**Figure 12:**
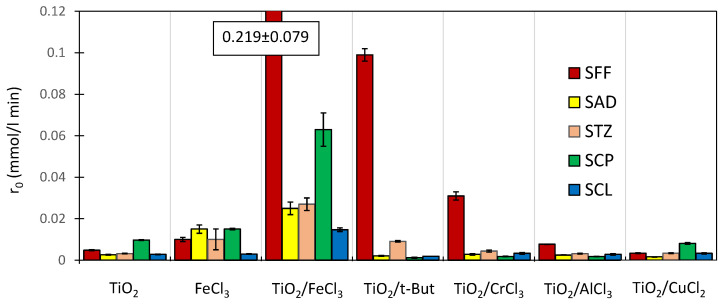
Photodegradation rates of SNs (0.1 mmol/L) in the presence of various catalyst systems at pH 3.0 ± 0.1.

In the case of two chlorine derivatives of SNs, i.e., sulfachlorpyridazine (SCP) and sulfaclozine (SCL), the concentration of the undissociated form in aqueous solution at pH 3 is similar to that of SFF (Table S3, Supplementary Materials). Therefore, SCP, SCL, SFF, SAD and STZ were used in experiments intended to assess the effects of N_amid_-C bond polarity and the concentrations of various molecular forms on the SNs photodegradation rate (0.1 mmol/L) in the presence of various catalyst systems (Figure 12).

#### 2.11.1. SNs Photodegradation in the Presence of TiO_2_ and/or FeCl_3_

In the presence of TiO_2_ alone (0.5 g/L) and in the presence of FeCl_3_ alone (1.0 mmol/L), SCP underwent the fastest photodegradation. However, the observed differences in the reaction rates of the studied SNs were not high (Figure 12). During the reaction performed in the presence of the TiO_2_/FeCl_3_ mixture, the photodegradation rates of all studied SNs were higher than the sums of the rates observed in reactions performed separately with TiO_2_ or FeCl_3_. Similar to SFF, SCP was also degraded at a high rate (r_0_ = 0.063 ± 0.008 mmol/L min) in the presence of a TiO_2_/FeCl_3_ mixture. Surprisingly, the photodegradation of SCL was slower than that of other SNs.

**Figure 13:**
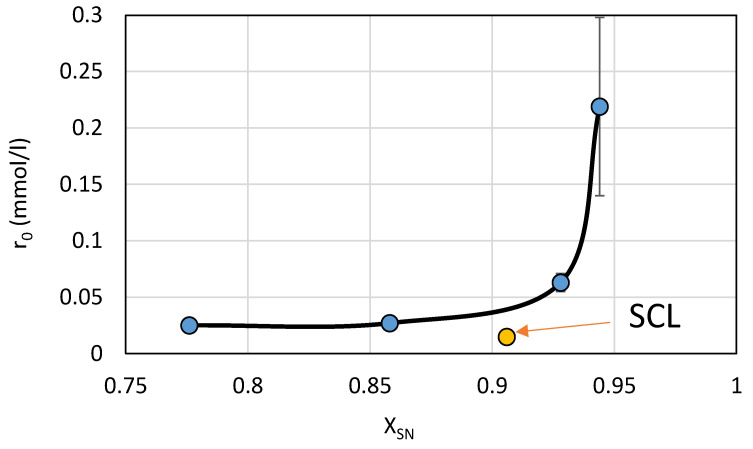
Relationship between the mole fraction of the molecular form of the SN (0.1 mmol/L) in aqueous solution at pH 3 and its photodegradation rate in the presence of TiO_2_/FeCl_3_.

These results indicate that the rate of photodegradation of the studied SNs, with the exception of SCL, in the presence of TiO_2_/FeCl_3_ increases with an increase in the concentration of the molecular form of SNs in aqueous solution at pH 3 (Figure 13). This result is consistent with our expectation that in this catalytic system, the concentration of the molecular form of SNs is an important determinant of the photodegradation rate.

The relationship between the degradation rate of the studied SNs and the pKa value is linear, with the exception of SCL (Figure S10, Supplementary Materials). The pK_a2_ values and the associated electron affinities of the amide substituents in SCP and SCL are very similar (Table S3, Supplementary Materials). Thus, despite the similarity of the structure of the amide substituent of SCL to that of the amide substituents of the other SNs, its photodegradation rate was much lower than expected (Figure 13). This can probably be attributed to the higher chemical stability of the 6-chloropyrazin-2-yl structure (Table S3, Supplementary Materials) and the higher stability of SCL to degradation in the presence of TiO_2_ or FeCl_3_ alone. In addition, this structure allows the formation of only one coordination bond involving a lone pair of electrons on the nitrogen atom with Fe^3+^ ion. The two nitrogen atoms in the 3-chloropyridazine ring of the SCP molecule are close to each other, and both can probably coordinate with Fe^3+^. This may affect the higher photodegradation rate of SCP in the presence of FeCl_3_ and TiO_2_/FeCl_3_.

#### 2.11.2. Photodegradation in the Presence of TiO_2_/t-But

The addition of t-But (10 mmol/L) increased the photodegradation rate of SFF and STZ at pH 3.0 ± 0.1 in comparison to the experiments performed with TiO_2_ alone (Figure 12). This positive effect disappeared with an increase in pH (Figure S11, Supplementary Materials). This may indicate that only neutral SFF and STZ molecules are reactive and can participate in the reaction. In addition, in the presence of TiO_2_/t-But, the photodegradation rates of SAD, SCP and SCL decreased.

In the reaction of t-But with HO^•^ radicals (Equation (31)), less active but long-lived C_4_H_8_OH^•^ radicals formed [58]. The results confirm that, unlike the degradation of other SNs, SFF degradation of neutral SFF molecules can be initiated by less active radicals (Equation (36)).

C_11_H_13_N_3_O_3_S + C_4_H_8_OH^•^ → C_11_H_12_N_3_O_3_S^•^ + C_4_H_9_OH (36)

SFF molecules, whether adsorbed or nonadsorbed, probably react with low-energy radicals generated during the photocatalytic reaction, e.g., with radicals formed in the reaction of SNs and other organic compounds with h^+^ (Equation (5)). These reactive molecules can be generated even from fatty acids, amino acids or sugars during photocatalytic reactions in wastewater. Thus, they probably react with SFF in propagation processes, leading to its degradation. This explains the high photodegradation rate of SFF in wastewater (Section 2.1). In turn, the inhibition of the degradation of other studied SNs (with the exception of STZ) in the presence of t-But indicates that C_4_H_8_OH^•^ does not initiate their degradation. It is also likely that active molecules generated from wastewater components do not initiate the degradation of other studied drugs (Figure 1). Degradation of the anionic form of SNs occurring at a higher pH (Figure S11, Supplementary Materials) was primarily initiated by HO^•^ radicals [59]. This was confirmed by the presence of hydroxyl derivatives among the products of SFF degradation in the presence of TiO_2_ at pH ~7 (Table 1). Therefore, a decrease in the supply of HO^•^ radicals consumed in competitive reactions inhibits photocatalytic degradation at a higher pH.

#### 2.11.3. Photodegradation in the Presence of TiO_2_/AlCl_3_, TiO_2_/CrCl_3_ and TiO_2_/CuCl_2_

The products of partial hydrolysis of Cr^3+^ and Al^3+^ cations and, to a lesser degree, Cu^2+^ affect the surface charge of TiO_2_ particles similarly to products of Fe^3+^ ion hydrolysis [60]. The Cr^3+^ ion can also form six coordination bonds, and the Cu^2+^ ion can form four (the complexing properties of Al^3+^ are much lower). Like Fe^3+^, Cr^3+^ cations can serve as electron donors and acceptors, while Cu^2+^ cations can only act as e^−^* acceptors. For these reasons, photocatalytic degradation of the studied SNs (0.1 mmol/L) in the presence of a mixture of TiO_2_ (0.5 g/L) with AlCl_3_, CrCl_3_ or CuCl_2_ salts (1.0 mmol/L) at pH 3.0 ± 0.2 was conducted. The results are presented in Figure 12.

The addition of CrCl_3_ caused an increase in the photodegradation rates of SFF and STZ. In the case of SFF, the r_0_ value increased almost 6-fold compared to the reaction conducted in the presence of TiO_2_ alone. The addition of AlCl_3_ caused a slight increase in the photodegradation rate of SFF only. In other experiments and always after the addition of CuCl_2,_ a decrease in the photodegradation rate was observed. These results indicate that e^−^* binding by Cu^2+^ ions did not increase the photodegradation rates of the studied SNs. Moreover, the addition of Al^3+^ salt and the resulting change in the surface charge of TiO_2_ (at pH 3) did not significantly increase the photodegradation rate of SNs. In turn, the positive effect of Cr^3+^ ions on SFF photodegradation can indicate that the ability to form multiple coordination bonds with a metal cation that is also redox amphoteric is important.

### 2.12. Influence of Steric Effect on SNs Binding

**Figure 14:**
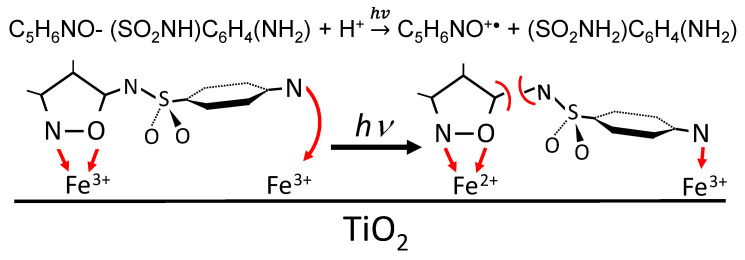
Schematic representation of the binding and light-initiated breaking of SFF molecules in the presence of TiO_2_/FeCl_3._ The red arrows indicate the direction of mass-transfer or charge-transfer.

Table 1 presents the spatial structures of all of the studied SNs. In the case of STZ, SCP and SKL, the heterocyclic substituent is positioned relative to the benzene ring at an angle of ~90° and the SFF molecule has an approximately linear structure. Therefore, only SFF can bind to the TiO_2_/FeCl_3_ system via its sulfonamide group (or via heteroatoms of the amide substituent) and via the amino group simultaneously (Figure 14). The resulting bond stress between the amide nitrogen and the heterocyclic substituent most likely facilitates its breaking.

This assumption can be confirmed by the presence of SAD and reaction products with the dimethylisoxazole radical in the solution after photocatalytic degradation of SFF (Section 2.2). The other studied SNs cannot simultaneously form coordination bonds with amine nitrogen and heteroatoms of the heterocyclic substituent. The SAD molecule has no heterocyclic substituent, while the spatial arrangement of the benzene ring relative to the heterocyclic substituent in STZ, SCP and SCL prevents the simultaneous formation of these bonds at both ends of the molecules. We assume that this is one of two main reasons for the large differences in the susceptibility of the studied SNs to photocatalytic degradation. However, the presented hypothesis has some limitations. The results of 3D structure analysis using in silico methods obtained by various procedures are often divergent (Table S3, Supplementary Materials) [20]. In addition, the hypothesis does not take into account the fact that water molecules can affect the spatial arrangement and the nature of SNs bonds [61]. It is also possible to create multiple bonds between Fe^3+^ and the oxygen in the sulfonamide group [46]. These additional bonds would change the spatial structure of SNs and the way of bonding of SNs molecules on the TiO_2_ surface.

### 2.13. Mechanism of SFF Photodegradation in the Presence of TiO_2_/FeCl_3_

**Figure 15:**
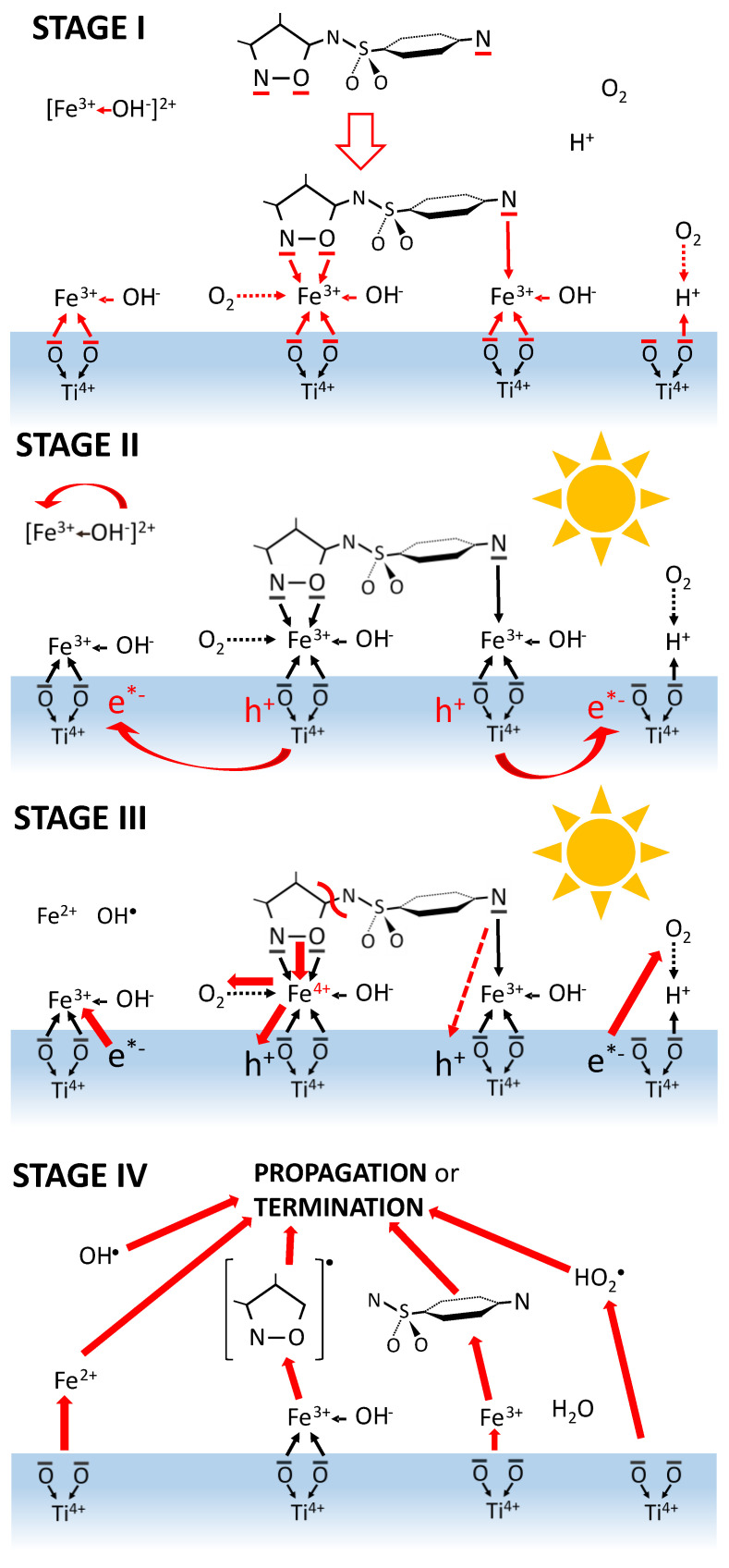
Stages of SFF photocatalytic degradation performed in the presence of TiO_2_/FeCl_3_. The red arrows indicate the direction of mass-transfer or charge-transfer.

Figure 15 presents the proposed SFF photodegradation mechanism in the presence of TiO_2_/FeCl_3_. The studied process can be divided into four stages. Stage I involves the diffusion of reagents to the TiO_2_ surface and the formation of coordination bonds with the oxygen atoms at active sites of the photocatalyst. In this way, H_3_O^+^, Fe(OH)^2+^ and [Fe(OH)(SFF)]^2+^ cations are adsorbed, while neutral molecules of SFF or O_2_ bind to the TiO_2_ surface via Fe^3+^ or H_3_O^+^ ions. Stage II concerns the processes that occur during irradiation of the reagents in the catalytic system with radiation of λ < 400 nm. Photoexcited h+/e^−^* pairs form on the TiO_2_ surface (Equation (1)) and they initiate charge flow between the catalyst surface and adsorbed compounds (Figure 15, stage III). Additionally, HO^•^ and HO_2_^•^ radicals form (Equations (2), (4) and (17)) at stage II. Fe^3+^ cations can also act as e^−^* acceptors and as an additional source of HO^•^ radicals (Equation (32)). However, we assume that the most important process at this stage is electron transfer from Fe^3+^ to h^+^ and the formation of an active complex containing Fe^4+^ (Equation (37)):

Fe^3+^ + h^+^ → Fe^4+^ (37)

Fe^4+^ can act as an electron acceptor from coordinated SNs molecules due to its high oxidizing potential (Figure 15, stage III). The charge transfer and the energy this process transfers to the SFF molecule can break a bond with the neighboring amide nitrogen, forming the SAD molecule and the C_5_H_6_NO^•+^ radical. Fe^2+^ cations form as a result of this electron transfer and form much weaker coordination bonds than Fe^3+^ ions. Therefore, the products are released from the TiO_2_ surface (Figure 15, stage IV). Desorbed particles, e.g., long-lived free radicals, can participate in propagation and termination reactions with each other, with HO^•^ radicals generated in solution and with not adsorbed molecules of reactants. The final effect of these processes is the total mineralization of organic compounds. It is possible that other processes occur in parallel with degradation in the irradiated mixture, but they do not have a significant effect on the SFF degradation rate in the presence of TiO_2_/FeCl_3_ at pH 3.

## 3. Materials and Methods

### 3.1. Reagents

The data on the SNs used in the experiments are listed in Table S3, Supplementary Materials. Solid TiO_2_ (Aeroxide^®^ P25, Degussa, Essen, Germany) was used as the photocatalyst. HCl, NaOH, FeCl_3_·6H_2_O, AlCl_3_·6H_2_O, CuCl_2_·2H_2_O and Na_2_SO_4_ (all analytical grade, POCH, Gliwice, Poland), tert-butanol (≥99.5%, Sigma-Aldrich, St. Louis, MO, USA), CrCl_3_·6H_2_O (≥98.0%, Merck, Darmstadt, Germany) and argon 5.0 (99.999%, Linde Gas, Eggendorf, Austria) were also used in the experiments. In the screening tests, sulfadiazine, sulfamethoxazole, sulfamethoprim, metronidazole, ifosfamide, 6-mercaptopurine, gentamicin, cefuroxime, doxycycline and ampicillin were used. These compounds were added at 0.1 mmol/L to synthetic wastewater prepared according to ISO 9887:1992(E) [62]. Detailed characteristics of the pharmaceuticals and synthetic wastewater components are provided in Tables S1 and S2.

### 3.2. Screening Tests

A total of 1 × 10^−5^ mol of each selected pharmaceutical was added to 100 mL of synthetic wastewater (Tables S1 and S2, Supplementary Materials). All of the samples were placed in open glass crystallizers (500 mL, exposed surface: 102 cm^2^), and solid TiO_2_ (50 mg) and FeCl_3_ solution (1 mL, 0.1 mol/L) were then added. The pH of the samples was adjusted to 3.0 ± 0.1 with HCl solution (1 mol/L). The pH of the samples was monitored using an HD22569.2 Multimeter (Delta OHM, Caselle di Selvazzano, Italy). After the addition of TiO_2_, all of the operations were conducted under protective (red) light. The samples in the crystallizers were mixed in the dark for 20 min and then irradiated using four fluorescent lamps (Actinic BL W/40, Philips, Amsterdam, The Netherlands). The intensity of radiation at the solution’s surface was measured using a quantum photoradiometer DO9721 (Delta OHM, Caselle di Selvazzano, Italy), and the wavelength λ < 400 nm was approximately 13.6 W/m^2^. During the experiments, the samples had free contact with air, and the temperature was 25 ± 2 °C.

### 3.3. Studies of the Kinetics of SFF Photocatalytic Degradation

100 mL of SFF solution (0.01–0.5 mmol/L) in redistilled water was placed in open glass crystallizers. Solid TiO_2_ (0–1 g/L) and/or an appropriate aliquot of a concentrated FeCl_3_ solution was added to this solution. The final concentration of FeCl_3_ in the samples ranged from 0.1 to 2.0 mmol/L. If necessary, the pH of the solution was adjusted by adding an appropriate aliquot of HCl or NaOH (1 mol/L) solution. After being stirred in the dark for 20 min, the samples were irradiated (0–60 min) under conditions identical to those described in Section 3.2.

### 3.4. Comparison of the Photodegradation Kinetics of SFF with Those of Other SNs

100 mL of a solution of each SN (0.1 mmol/L) in redistilled water (Table 1) was placed in open glass crystallizers. Solid TiO_2_ (0.5 g/L) and/or 1 mL of a freshly prepared FeCl_3_ solution (1.0 mol/L) and/or 1 mL of a solution of a compound that acts as a selective initiator or inhibitor were added to the SNs solutions (Table S5, Supplementary Materials). If necessary, the pH of the solution was adjusted by adding an appropriate aliquot of HCl or NaOH (1 mol/L) solution. After being stirred in the dark for 20 min, the samples were irradiated (0–60 min) under conditions identical to those described in Section 2.2.

### 3.5. Comparison of the Kinetics of SNs Photodegradation under Aerobic and Anaerobic Conditions

A schematic diagram of the test stand used to irradiate the samples under aerobic and anaerobic conditions is shown in Figure S12 (Supplementary Materials). Open 250 mL beakers containing 150 mL of a solution of SAD, SFF or STZ (0.1 mmol/L) in redistilled water were ventilated for 15 min with compressed air or argon. The concentration of dissolved oxygen in each solution was monitored using a multimeter HD22569.2 outfitted with a probe (D097099S, Delta OHM, Caselle di Selvazzano, Italy). Then, under protective (red) light and without interrupting the gas flow, the selected catalyst system was added to the solutions. After an additional 15 min of ventilation in the dark, the samples were irradiated using four fluorescent lamps (Actinic BL W/40, Philips, Amsterdam, The Netherlands). The radiation intensity measured at the solution’s surface was determined to be approximately 13.6 W/m^2^.

### 3.6. UPLC Analysis

The samples (~2.5 mL) that were collected before and during irradiation were centrifuged immediately (10 min, 4000 RPM). The concentration of SNs in the resulting supernatants was determined by UPLC (ACQUITY UPLC I-Class System, Waters Corp., Milford, MA, USA). An ACQUITY UPLC BEH C18 column (130Å, 1.7 µm, 2.1 mm × 100 mm) was used at a temperature of 35 °C with sample volumes of 1 µL and 5 µL. Mobile phase A was H_2_O (LC-MS grade, LiChrosolv^®^, Sigma-Aldrich, St. Louis, MO, USA) containing 0.01% HCOOH (98–100% for LC-MS LiChropur^®^, Supelco, Bellefonte, PA, USA), and mobile phase B was CH_3_CN (hypergrade for LC-MS LiChrosolv^®^, Sigma-Aldrich, St. Louis, MO, USA) containing 0.01% HCOOH. Detailed data on the UPLC analysis are included in Table S6. PDA (λ = 272 nm) and Xevo G2-XS QTof, ESI^+^ detectors (Waters Corp., Milford, MA, USA) were used.

### 3.7. Fe^3+^ Ions Determination

The Fe^3+^ concentration in the supernatants was determined spectrophotometrically. A total of 1 mL of NH_4_SCN solution (20%; analytical grade, POCH, Gliwice, Poland) in redistilled water and 4 mL of HCl solution (0.1 mol/L) were added to 1 mL of supernatant. The absorbance of the samples relative to that of a sample without Fe^3+^ compounds was measured within 5 min (λ = 480 nm, 1 = 1 cm) using a spectrophotometer (DR 3900, HACH, Düsseldorf, Germany). Method validation was performed using a solution of NH_4_Fe(SO_4_)_2_·12H_2_O (analytical grade, POCH, Gliwice, Poland).

### 3.8. Identification of SNs Photodegradation Products

The products of SNs photodegradation were identified based on a comparison of chromatograms (QTof detector, ESI+) of non-irradiated and irradiated samples (Figure S1, Supplementary Materials). The compounds resolved on the chromatograms prior to the irradiation of the samples were not identified. Monoisotope masses (M + H^+^) and fragmentation spectra, which were obtained at fragmentation energies in the range of 10–25 V, were determined for peaks corresponding to the degradation products (Figure S13, Supplementary Materials). Chemical structures were drawn using ChemDraw Std software with the Analysis package (CambridgeSoft). The identification of labile amides and peroxides was not possible due to the type of ion source (ESI+) and the type of eluent.

### 3.9. Total Organic Carbon Analysis

The Total Organic Carbon (TOC) removal in the SNs solutions before and after UV irradiation was performed using the LCK380 cuvette test (HACH LANGE, Düsseldorf, Germany), and the results were read on a DR 3900 spectrophotometer (HACH LANGE, Düsseldorf, Germany).

### 3.10. Analysis of Results

Graphs of the function *C/C*_0_ = *f*(*t*), where *C* is the reactant concentration after irradiation time (*t*) and *C*_0_ is the initial reactant concentration, were prepared based on the results of UPLC analysis. The photocatalytic processes that occur in the presence of TiO_2_ or FeCl_3_ are believed to be pseudo-first-order reactions [16,20,36,53]. Therefore, the value of the reaction rate constant (*k*) was determined from the slope of the linear equation (Equation (38)):

*ln*(*C*_0_/*C*) = *kt* + *b* (38)

where *b* is the intercept.

The initial reaction rate (*r*_0_) was determined using Equation (38):

*r*_0_ = *kC*_0_ (39)

The degree of adsorption, binding or removal (*a*) was determined according to the following equation (Equation (40)):

*a* = 100 · (1 − (*C_eq_*/*C*_0_)) (40)

where *C_eq_* is the equilibrium concentration of SN or Fe^3+^ in the solution after the addition of the catalytic system components (i.e., after adsorption but prior to irradiation).

## 4. Conclusions

In aqueous solutions, SFF photodegradation in the presence of a TiO_2_/FeCl_3_ mixture was found to occur many times faster than the photodegradation of other pharmaceuticals. The kinetics of this process correlates with the L–H heterogeneous catalysis model, and the optimum pH is sharp at approximately 3.

In the abovementioned catalytic system, Fe(OH)^2+^ ions adsorb onto the TiO_2_ surface and simultaneously form complexes with SFF. Neutral SFF molecules coordinated with Fe^3+^ ions are primarily involved in the photocatalytic process. The products of hydrolysis or polymerization of Fe(OH)^2+^ ions inhibit SFF photodegradation.

The amount of SFF adsorbed on TiO_2_/FeCl_3_ at equilibrium is low (<10%), and the resulting complexes are probably labile. The possibility that the SFF photodegradation rate depends on the adsorption rate and not on the adsorption equilibrium cannot be excluded.

The identified products of SFF photodegradation in the presence of TiO_2_/FeCl_3_ are SAD and dimethylisoxazole group adducts. These compounds form as a result of the breakage of the amide bond in the SFF molecule, with the simultaneous formation of dimethylisoxazole radical or cation radicals. Surprisingly, among the SFF photodegradation products, there are no hydroxyl derivatives that are characteristic of analogous reactions conducted in the presence of TiO_2_ or FeCl_3_ and initiated by HO^•^ radical. The photodegradation rates of all studied SNs in the presence of TiO_2_/FeCl_3_ are much higher than the sums of the photodegradation rates observed in reactions performed in the presence of TiO_2_ or FeCl_3_ individually.

During photodegradation of SNs in the presence of TiO_2_/FeCl_3_, reduction of Fe^3+^ ions occurred in parallel. The rate of this reaction was lower than the SFF photodegradation rate but higher than the photodegradation rates of other SNs.

The factor that determines the rate of SFF photodegradation is probably the charge transfer from the coordinated reactant to h^+^ mediated by Fe^3+^ ions. An active complex containing Fe^4+^ and oxygen, as well as long-lived free radicals that participate in the degradation of subsequent non-adsorbed SFF molecules, can form at this stage. Other reactions, including e^−^* binding by adsorbed Fe^3+^ compounds, may also occur in parallel.

The presence of other complex-forming Me^3+^ cations that possess oxidation/reduction properties (redox amphoteric) can also, but to a lesser extent, accelerate the SFF photodegradation rate in the presence of TiO_2_. SO_4_^2−^ ions limit SFF adsorption to the TiO_2_ surface and inhibit photodegradation.

Of the studied SNs, SFF exhibited the highest rate of photodegradation. That effect may be caused by one or more of the following:

- The high bond polarity between the amide nitrogen and the heterocyclic substituents;
- The nearly linear structure of the SFF molecule;
- The specific arrangement of heteroatoms in the heterocyclic moiety, allowing the formation of a chelated complex;
- A high molar fraction of the molecular form in solution at the optimal pH for photocatalytic activity;
- Susceptibility to initiation of the degradation process by low-active, long-lived radicals.

The limitation of SFF photodegradation is the narrow range of the optimal pH value. The improvement of the degradation efficiency of drugs present in large amounts in post-culture wastewater can be achieved by using drugs with a high, selective susceptibility to photocatalytic degradation in animal breeding. The results obtained indicate the feasibility of this solution.

## Figures and Tables

**Table 1:** Products of the photocatalytic transformation of selected SNs (0.1 mmol/L) conducted in the presence of TiO_2_/FeCl_3_, TiO_2_ and FeCl_3_.

SNs Catalytic System pH	t_R_^(1)^ (min)	Detected Mass M + H^+^ (Da)	Proposed Molecular Formula	Calculated Mass M + H^+^ (Da)	Proposed Structural Formula ^(2)^
SFF TiO_2_/FeCl_3_ pH 3.0	0.98	140.1073	C_8_H_13_NO	140.1075	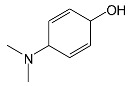
	1.37	174.0226 ^(3)^	C_6_H_8_N_2_O_2_S	174.0225	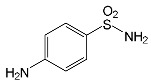
	1.74	196.1084	C_9_H_13_N_3_O_2_	196.1086	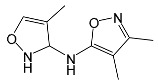
	2.10	199.1078	C_9_H_14_N_2_O_3_	199.1083	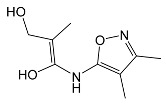
	2.45	153.1026	C_8_H_13_N_2_O	153.1028	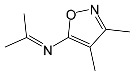
	3.38	353.1278	C_15_H_21_N_4_O_4_S	353.1284	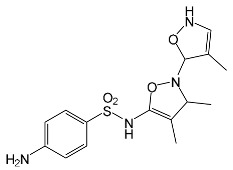
	5.62	379.1069	C_16_H_18_N_4_O_5_S	379.1076	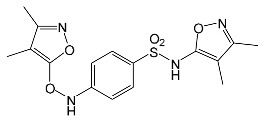
	6.08 6.55	533.1275	C_22_H_24_N_6_O_6_S_2_	533.1277	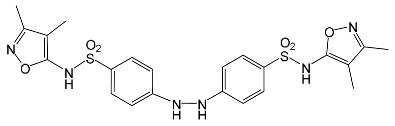
	6.94	492.1006	C_20_H_21_N_5_O_6_S_2_ ^(3)^	492.1011	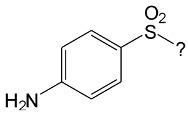 ^(4),(5)^
SAD TiO_2_/FeCl_3_ pH 3.0	2.52 2.58	262.0063	C_12_H_11_N_3_O_2_S	262.0061	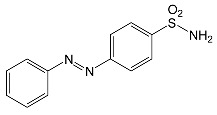
	2.64	276.0446	C_12_H_9_N_3_O_3_S	276.0443	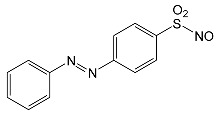
	2.98 3.22	357.0325	C_12_H_12_N_4_O_5_S_2_	357.0327	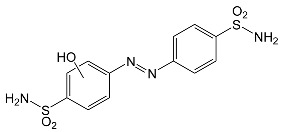
	3.42	263.0488	C_12_H_10_N_2_O_3_S	263.0490	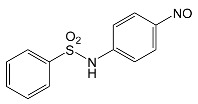
	3.58	511.0535	? ^(4)^	?	?
	3.66	433.0626	C_18_H_16_N_4_O_5_S_2_ ?	433.0640	?
STZ TiO_2_/FeCl_3_ pH 3.0	0.49	101.0176	C_3_H_4_N_2_S	101.0173	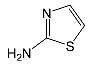
	1.91	168.0594	C_7_H_9_N_3_S	168.0595	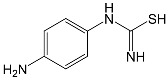
	2.22	290.0269	C_9_H_11_N_3_O_4_S_2_	290.0269	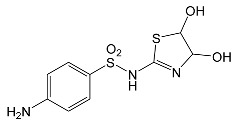
	2.61	196.0541	C_8_H_9_N_3_OS	196.0545	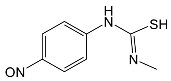
	3.68	346.0316	C_15_H_11_N_3_O_3_S_2_	346.0317	?
SFF TiO_2_ pH 7.5	1.29	189.0331	C_6_H_8_N_2_O_3_S	189.0334	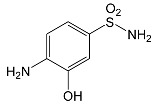
	1.37	174.0226 ^(3)^	C_6_H_8_N_2_O_2_S	174.0225	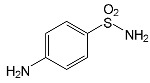
	3.52	284.0705	C_11_H_14_N_3_O_4_S	284.0705	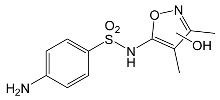
	3.88	298.0497	C_11_H_11_N_3_O_5_S	298.0498	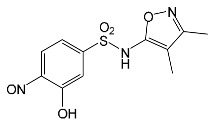
	3.94	300.0652	C_11_H_11_N_3_O_5_S	300.0654	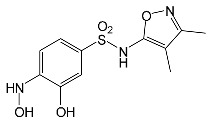
	4.92	284.0705	C_11_H_14_N_3_O_4_S	284.0705	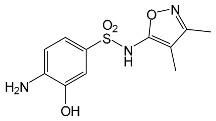
	6.35	387.0756	C_17_H_14_N_4_O_5_S ?	387.0763	?
	6.37	392.1025	C_16_H_17_N_5_O_5_S ?	392.1029	?
SFF FeCl_3_ pH 3.0	1.37	174.0226 ^(3)^	C_6_H_8_N_2_O_2_S	174.0225	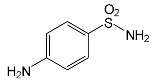
	2.10	199.1078	C_9_H_14_N_2_O_3_	199.1083	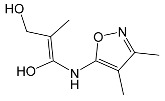
	2.52	113.0720	C_5_H_8_N_2_O	113.0715	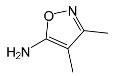
	5.01	285.0547	C_11_H_12_N_2_O_5_S	285.0545	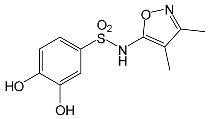
	5.86	452.0692	C_17_H_18_N_5_O_6_S_2_	452.0698	?
	6.80	563.1017	C_22_H_22_N_6_O_8_S_2_	563.1019	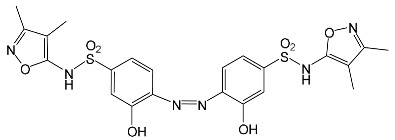
	6.97	638.1514	?		?

^(1)^ Retention time on the chromatograms (Figure S1, Supplementary Materials); ^(2)^ based on the monoisotopic masses (M + H^+^) and fragmentation spectra; ^(3)^ compound identified based on the tR of the standard; ^(4)^ (?) structure was not identified; ^(5)^ a substituent of the sulfonic group was not identified.

## Data Availability

The data presented in this study are available on request from the corresponding author. The data are not publicly available due to very large sizes of chromatographic files.

## Supplementary Materials

The following are available online at https://www.mdpi.com/article/10.3390/ijms22168696/s1. References [63,64,65,66,67,68,69,70,71,72,73,74,75,76,77,78,79,80,81,82,83,84,85,86,87,88,89,90] are cited in the supplementary materials.
