# Supplementary material for: A New Mechanism of the Selective Photodegradation of Antibiotics in the Catalytic System Containing TiO2 and the Inorganic Cations
Source: Int J Mol Sci. 2021 Aug 13;22(16):8696. doi: 10.3390/ijms22168696 (PMC8395856; doi:10.3390/ijms22168696)
Supplement: Supplementary file 1 [file ijms-22-08696-s001.zip › ijms-1336702-supplementary.pdf]

International Journal of
Molecular Sciences

Article

A New Mechanism of the Selective Photodegradation of Antibiotics in the Catalytic System Containing TiO_2 and the Inorganic Cations

Wojciech Baran [1], Mateusz Cholewiński [1], Andrzej Sobczak [1] and Ewa Adamek [1,*]

[1] Department of General and Analytical Chemistry, Faculty of Pharmaceutical Sciences in Sosnowiec, Medical University of Silesia in Katowice; Jagiellońska 4, 41-200 Sosnowiec, Poland; wbaran@sum.edu.pl (W.B.); mateochol@tlen.pl (M.C.); asobczak@sum.edu.pl (A.S.); eadamek@sum.edu.pl (E.A);
* Correspondence: e-mail: eadamek@sum.edu.pl; tel: +48 32 364 15 65

Tables and figures list in Supplementaty Materials

Table S1. Characteristics of the investigated pharmaceuticals used in screening tests.

Table S2. Chemical composition of the synthetic municipal wastewater (ISO 9887:1992(E)).

Table S3. Characteristics of the investigated SNs

Table S4. Characteristics of the radicals and cations

Table S5. Concentrations of substances used in studies of the photocatalytic degradation of SNs

Table S6. Detailed data for the UPLC procedure (gradient table)

Figure S1a. TOF/BPI chromatograms of SFF solution irradiated for 0, 30 and 90 s in the presence of a $TiO_2/FeCl_3$ mixture.

Figure S1b. TOF/BPI chromatograms of SFF solution irradiated for 15 and 40 min separately in the presence of TiO_2 or $FeCl_3$.

Figure S1c. TOF/BPI chromatograms of SAD solution irradiated for 2 and 10 min in the presence of a $TiO_2/FeCl_3$ mixture.

Figure S1d. TOF/BPI chromatograms of STZ solution irradiated for 2 and 10 min in the presence of a TiO2/FeCl3 mixture

Figure S2. Changes in the concentration of SFF solution in the presence of a mixture of TiO_2 (0.5 g/l)/$FeCl_3$ (1.0 mmol/l) in the dark and irradiated with UVA

Figure S3. Effect of pH on the photodegradation rate of SFF (0.1 mmol/l) in the presence of a TiO_2 (0.5 g/l)/$FeCl_3$ (1.0 mmol/l) mixture

Figure S4. Effect of the initial concentration of SFF on the degree of sorption (before UVA irradiation) in the presence of a TiO_2 (0.5 g/l)/$FeCl_3$ (1.0 mmol/l) mixture

Figure S5. Effect of the amount of TiO_2 on the sorption of SFF (0.1 mmol/l) in the presence of $FeCl_3$ (1.0 mmol/l).

Figure S6. Changes in the concentration of SNs (C_0 0.1 mmol/l) and Fe^{3+} (C_0 1.0 mmol/l) during the photodegradation conducted in the presence of a $TiO_2/FeCl_3$ mixture at pH 3.0±0.1.

Figure S7. Comparison of the degradation rate constants od the selected SNs (0.1 mmol/l) during the Fenton process (0.15 mmol $FeSO_4$/l and 0.5 mmol H_2O_2/l) and Fenton-like reactions (photo Fenton, 1.0 mmol $FeCl_3$/l and 2.0 mmol H_2O_2/l)

Figure S8. Effect of the SO_4^{2-} concentration on the sorption of SFF (0.1 mmol/l) on the catalytic system (0.5 g TiO_2/l /1.0 mmol $FeCl_3$/l) at pH 3.0±0.1

Figure S9. Effect of the pH on the dissociation and the concentration of molecular form of SNs (0.1 mmol/l) in aqueous solution: cationic form (SH_2^+) – solid line, neutral form (SH) – dashed line, anionic form (S^-) – dotted line

Figure S10. Relationship between the photodegradation rate of SNs (0.1 mmol/l) in the presence of a TiO_2(0.5 g/l)/$FeCl_3$(1.0 mmol/l) mixture and their dissociation constants (K_a)

Figure S11. Effect of the pH on the initial rate of SFF photodegradation (0.1 mmol/l) in the presence of TiO_2 (0.5 g/l) or a TiO_2 (0.5 g/l)/t-But (10 mmol/l) mixture

Figure S12. Test stand for the photocatalytic experiments under aerobic and anaerobic conditions, (a) UVa lamps; (b) a glass beaker (5000 ml); (c) a glass beaker (250 ml) (d) SN solution (150 ml, 0.1 mmol/l) with the selected catalyst system; (e) a glass tube (drain)

Figure S13. MSMS spectrum and a possible formula of compound with m/z 196.1086 Da occurred in SFF solution after irradiation for 90 s in the presence of a $TiO_2/FeCl_3$ mixture

Table S1. Characteristics of the investigated pharmaceuticals used in screening tests.

Name, CAS number	Manufacturer	Chemical purity	Structural formula[1)]
Sulfanilamide<br>63-74-1	Sigma-Aldrich	>98.0%	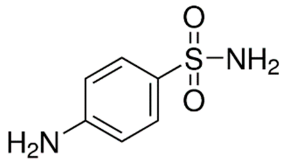
Sulfadiazine sodium salt<br>547-32-0	Sigma-Aldrich	≥98.0%	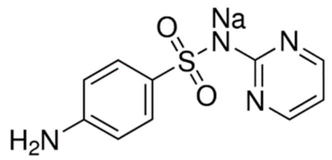
Sulfathiazole<br>72-14-0	Sigma-Aldrich	≥99.0%	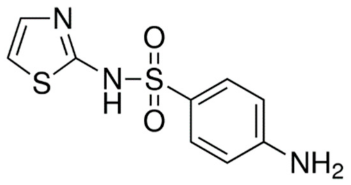
Sulfamethazine<br>57-68-1	Sigma-Aldrich	≥99.0%	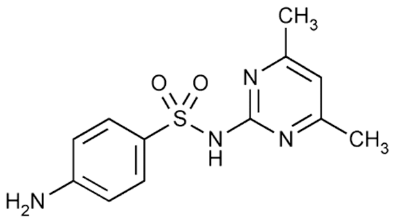 [2)]
Sulfamethoxazole<br>723-46-6	Sigma-Aldrich	≥99.0%	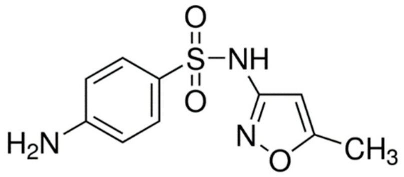
Sulfisoxazole<br>127-69-5	Sigma-Aldrich	≥99.0%	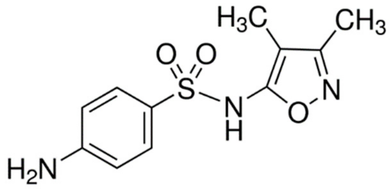
Trimethoprim<br>738-70-5	Sigma-Aldrich	≥99.0%	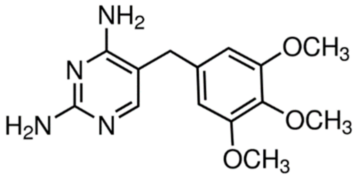
Metronidazole<br>443-48-1	Supelco		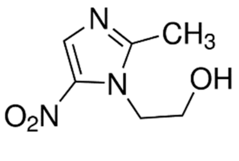

Ifosfamide<br>3778-73-2	Sigma-Aldrich	≥98.0%	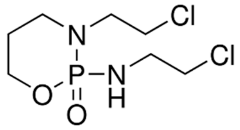
6-Mercaptopurine monohydrate<br>6112-76-1	Sigma-Aldrich	98.0%	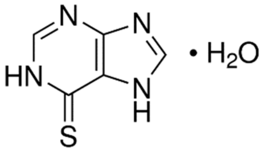
Gentamicin sulfate salt<br>1405-41-0	Sigma-Aldrich	≥590 µg gentamicin base per mg	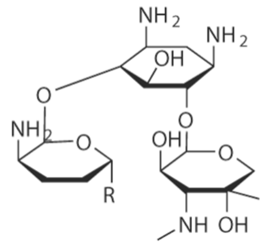 3)
Cefuroxime sodium salt<br>56238-63-2	Supelco		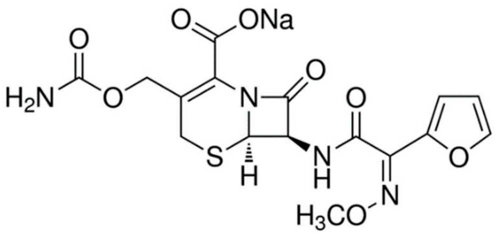
Doxycycline hyclate<br>24390-14-5	Sigma-Aldrich	≥93.5%	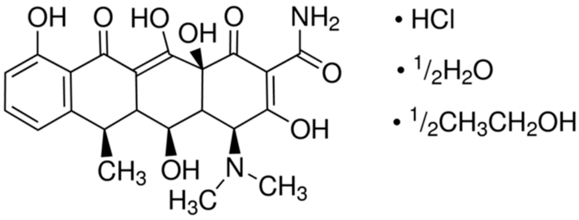
Ampicillin sodium salt<br>69-52-3	Sigma-Aldrich	≥98%	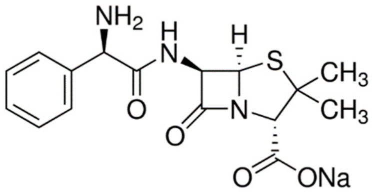

1) from the manufacturer's website

2) [63]

3) [64]

Table S2. Chemical composition of the synthetic municipal wastewater (ISO 9887:1992(E)) [62]

Chemicals	Manufacturer	Chemical purity	Concentration (mg/l)
Peptone from animal tissue	Sigma-Aldrich	-	160
Meat extract	Millipore	-	110
Urea	POCH (Poland)	p.a.	30
K_2HPO_4	POCH (Poland)	p.a.	28
NaCl	POCH (Poland)	p.a.	7
$CaCl_2{\cdot}2H_2O$	POCH (Poland)	p.a.	4
$MgSO_4{\cdot}7H_2O$	Chempur (Poland)	p.a.	2

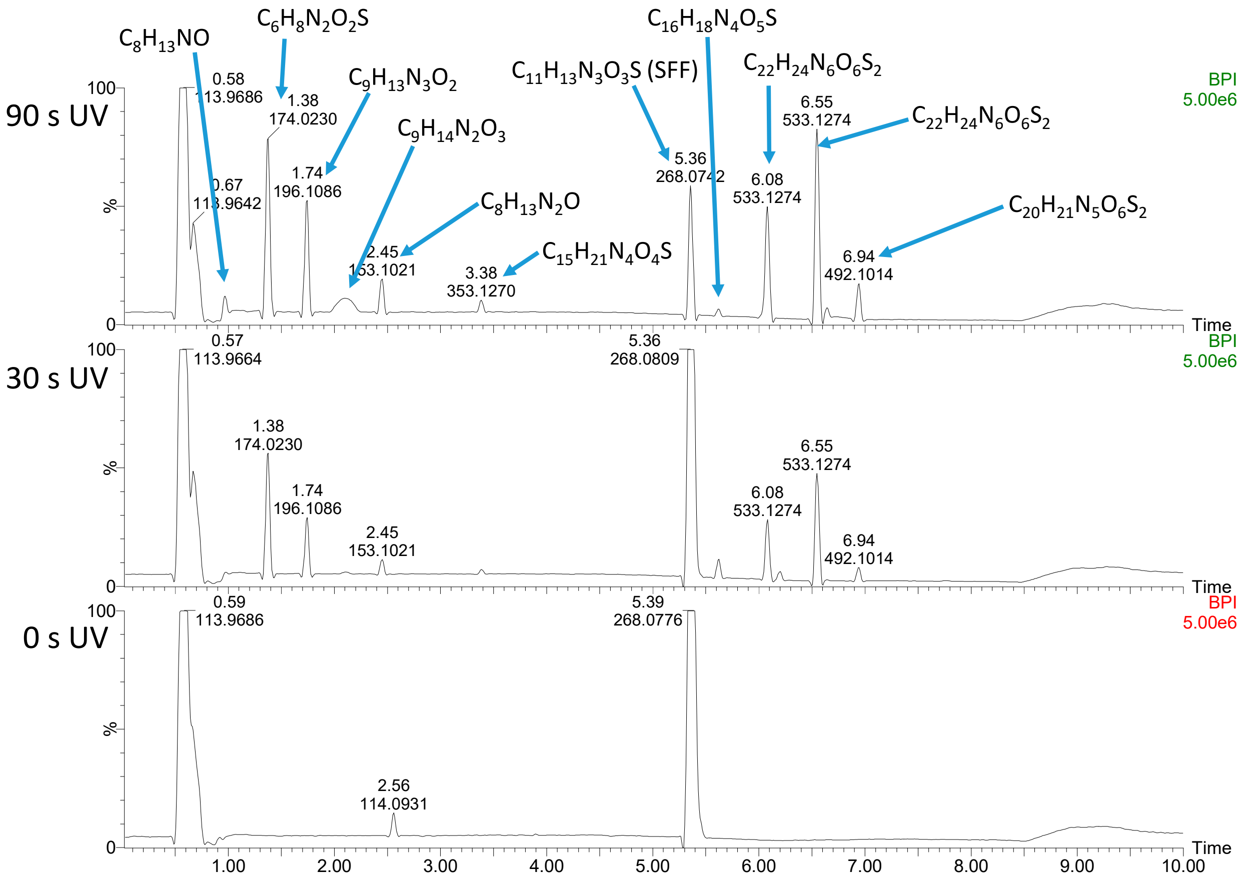

Figure S1a. TOF/BPI chromatograms of SFF solution irradiated for 0, 30 and 90 s in the presence of a $TiO_2/FeCl_3$ mixture

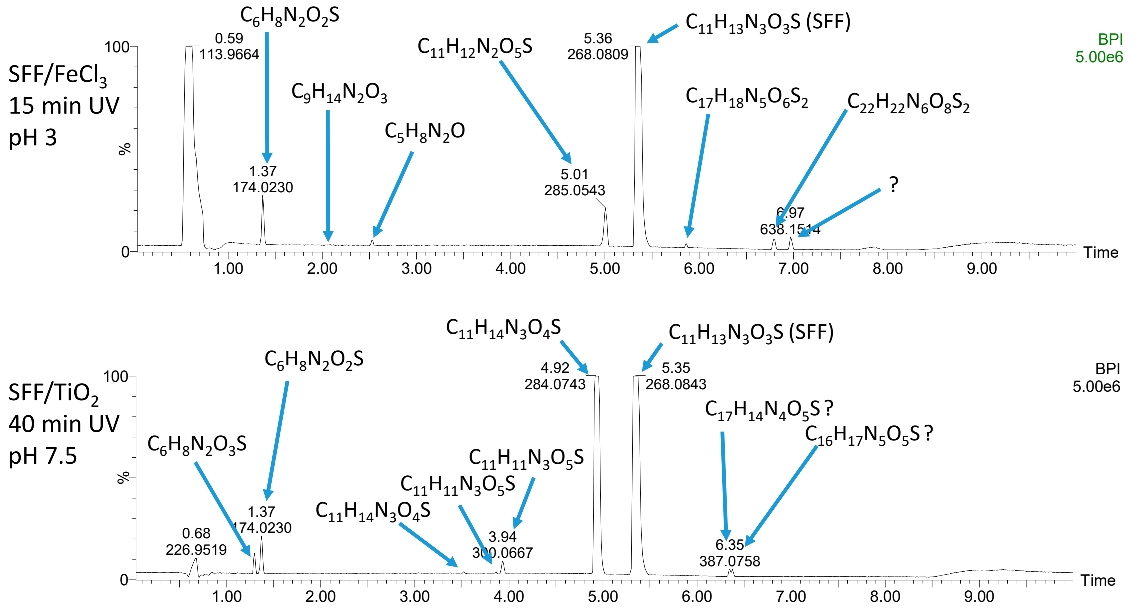

Figure S1b. TOF/BPI chromatograms of SFF solution irradiated for 15 and 40 min separately in the presence of TiO_2 or $FeCl_3$

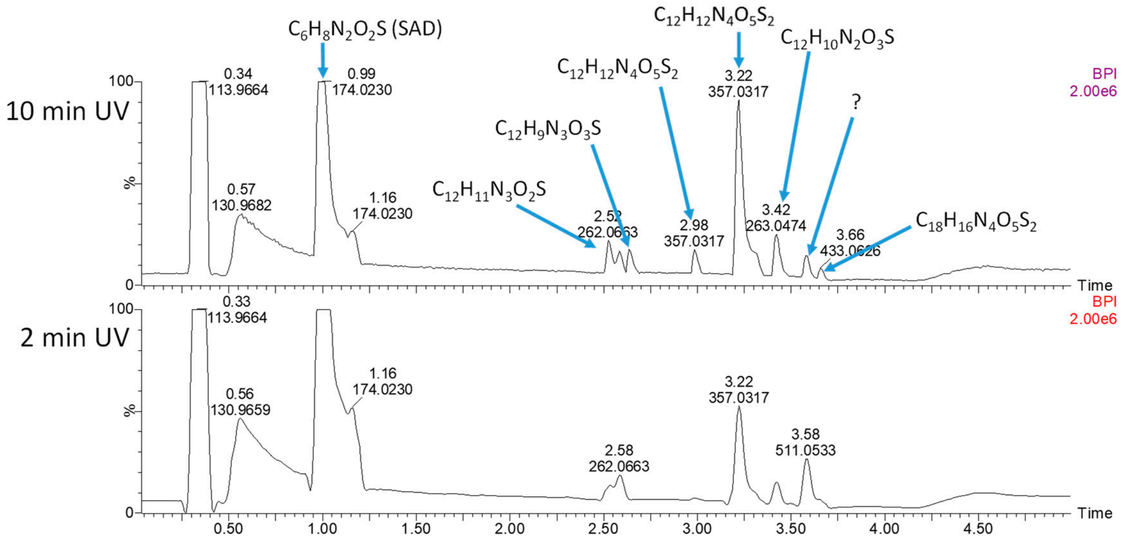

Figure S1c. TOF/BPI chromatograms of SAD solution irradiated for 2 and 10 min in the presence of a TiO_2/$FeCl_3$ mixture

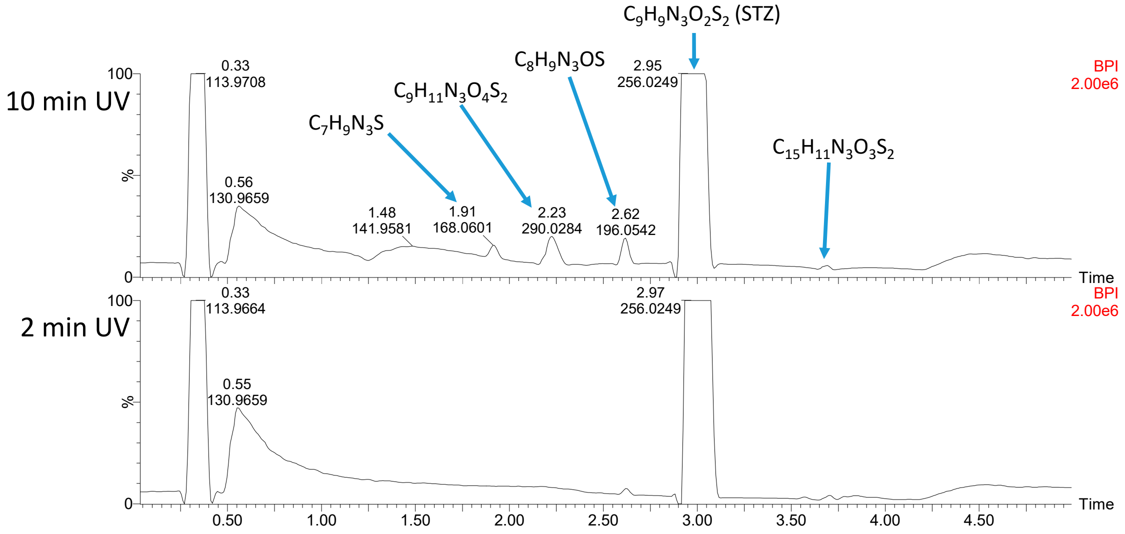

Figure S1d. TOF/BPI chromatograms of STZ solution irradiated for 2 and 10 min in the presence of a $TiO_2/FeCl_3$ mixture

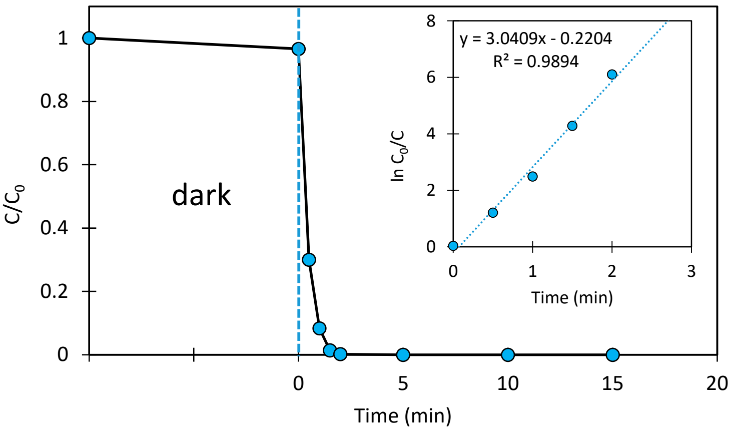

Figure S2. Changes in the concentration of SFF solution in the presence of a mixture of TiO_2 (0.5 g/l)/$FeCl_3$ (1.0 mmol/l) in the dark and irradiated with UVA

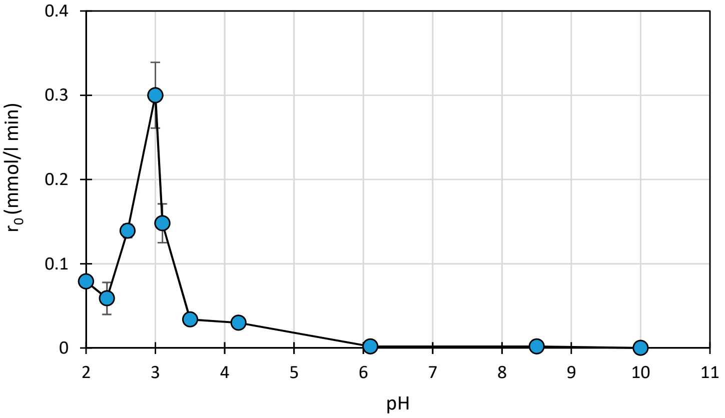

Figure S3. Effect of pH on the photodegradation rate of SFF (0.1 mmol/l) in the presence of a TiO_2 (0.5 g/l)/$FeCl_3$ (1.0 mmol/l) mixture

Table S3. Characteristics of the investigated SNs

Abbreviation	3D structures [65]	pK_{a1}	Molar fraction

Name,<br>CAS,<br>manufacturer,<br>chemical purity	in the text		pK_{a2} 1)	of the molecular form<br>(SH) 1)<br>at pH 3
Sulfanilamide<br>63-74-1<br>Sigma-Aldrich,<br>>98.0%	**SAD**	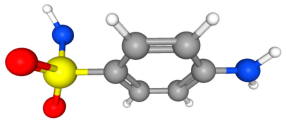	2.46 ± 0.12<br>10.35 ± 0.21 [20]	0.776
Sulfisoxazole<br>127-69-5<br>Sigma-Aldrich,<br>≥99.0%	**SFF**	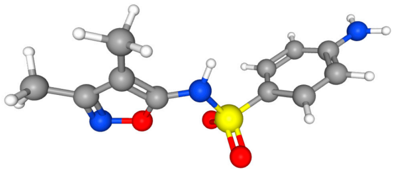	1.69 ± 0.27<br>5.00 ± 0.00 [20]	0.944
Sulfathiazole<br>72-14-0<br>Sigma-Aldrich,<br>≥99.0%	**STZ**	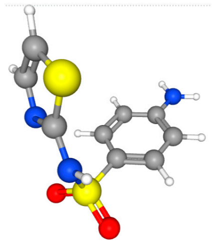	2.22±0.27<br>7.15±0.12 [20]	0.858
Sulfachlorpyridazine<br>80-32-0<br>Sigma-Aldrich,<br>99.7%	**SCP**	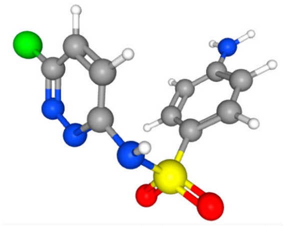	1.87±0.30<br>5.45±0.06 [66]	0.928
Sulfaclozine<br>23307-72-4<br>Sigma-Aldrich,<br>99.8%	**SCL**	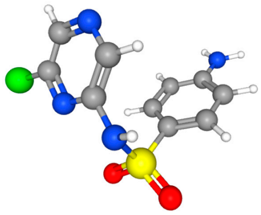	~2<br>5.5 [28]	0.906

1) $SH_2^+ \xleftrightarrow{Ka_1} SH \xleftrightarrow{Ka_2} S^-$,

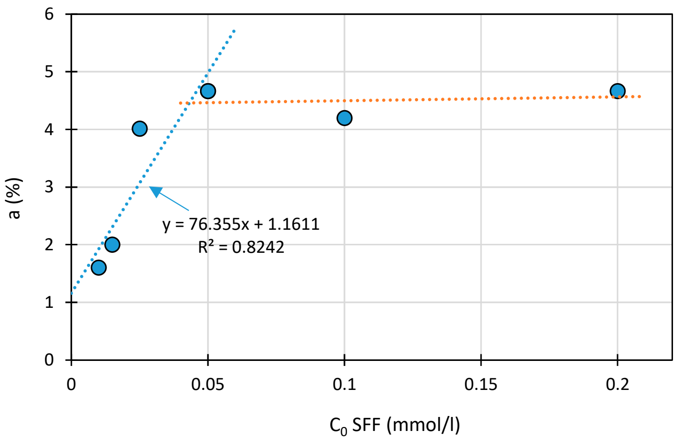

Figure S4. Effect of the initial concentration of SFF on the degree of sorption (before UVA irradiation) in the presence of a TiO_2 (0.5 g/l)/$FeCl_3$ (1.0 mmol/l) mixture

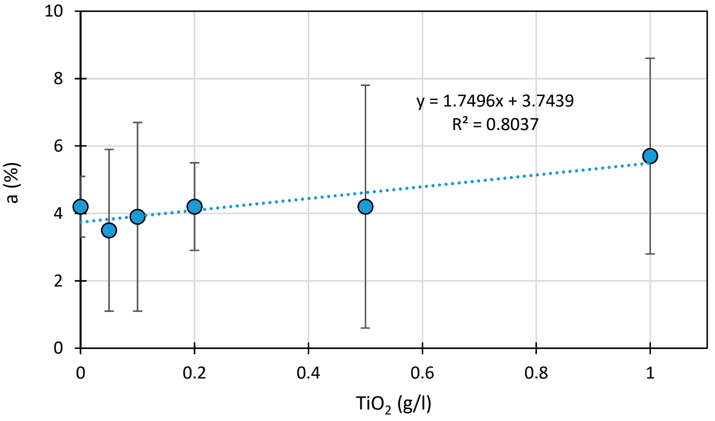

Figure S5. Effect of the amount of TiO_2 on the sorption of SFF (0.1 mmol/l) in the presence of $FeCl_3$ (1.0 mmol/l)

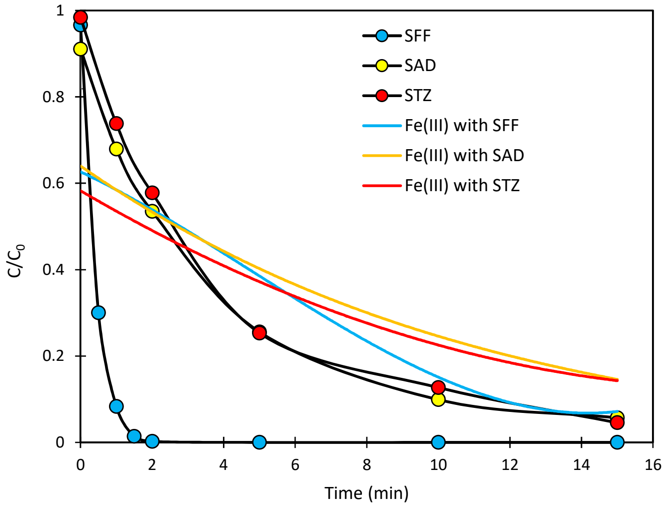

Figure S6. Changes in the concentration of SNs (C_0 0.1 mmol/l) and Fe^{3+} (C_0 1.0 mmol/l) during the photodegradation conducted in the presence of a TiO_2/$FeCl_3$ mixture at pH 3.0±0.1.

Table S4. Characteristics of the radicals and cations [1)]

Radical	Chemical name	Redox potential	Recombination	Reaction constant rate

		(V)	constant rate (mol/l s)	with other compounds (mol/l s)
$HO^{\bullet}$	hydroksyl	2.7 [67]	4.7×10^{9} [68]	9.7×10^{8} (methanol) [45]
		2.8 [69]	5.5×10^{9} [45]	7.9×10^{9} (benzene) [45]
		2.33 [70]	6×10^{9} [43]	3.1×10^{9} 2-butanol [45]
		2.34 [71]	5.2×10^{9} [72]	1.6×10^{7} (acetic acid) [45]
			5.3×10^{9} [73]	8.6×10^{9}(aniline) [74]
			4.2×10^{9} [75]	8.2×10^{9} (sulfanilic acid) [74]
				1.6×10^{8} (pyrimidine) [74]
				3.5×10^{9} (isoxazole) [74]
				$\leq 3.5 \times 10^{9}$ (SNs) [74]
				$0.66 - 1.8 \times 10^{10}$ (phenol) [75]
				$7.8 - 8.5 \times 10^{9}$ (SNs) [76]
				5.5×10^{9} (SMX) [77]
				$10^{9} – 10^{10}$ (lipid) [78]
				$2.8 \times 10^{9} - 3.4 \times 10^{10}$ (aliphatic alcohols) [79]
				2.7×10^{7} (H_2O_2) [80]
$O_2^{\bullet -}$	superoxide radical	0.94 [70,71]]	2.33×10^{6} [72]	$< 10^{2}$ [71]
		-0.33 [67,71]		$10^{5} - 10^{9}$ (ascorbate) [71]
				1.0×10^{9} (Fe^{3+}) [43]
$HO_2^{\bullet}$	hydroperoxyl	1.48 [71]	$(8.3 \pm 0.7) \times 10^{5}$ [45]	$1.18 - 3.05 \times 10^{3}$ (fatty acids) [71]
		1.7 [81]	1.8×10^{6} [43]	$10^{9} – 10^{10}$ (lipids) [78]
			2.33×10^{6} [72]	
			8.5×10^{5} [73]	
$Cl^{\bullet}$	chlorine radical	2.5 [45,79]	$1.35 \cdot 10^{7}$	1.0×10^{9} (methanol) [45]
		1.36 [44]	8.8×10^{7} [45]	5.0×10^{9} (butanol 2) [45]
				2.0×10^{8} (acetic acid) [45]
				9.0×10^{9} (benzene) [45]
				2.5×10^{8} (phenol) [45]
				9.4×10^{2} (SMX) [77]
$SO_4^{\bullet -}$	sulfate radical anion	2.60 – 3.10 [79]	7.0×10^{8} [45]	$5.6 \times 10^{6} - 9.9 \times 10^{7}$ (alkans) [79]
			$(6.1 \pm 0.15) \times 10^{8}$ [82]	$5.9 \times 10^{7} - 1.4 \times 10^{9}$ (aliphatic alcohols) [79]
$CH_2OH^{\bullet}$	hydroxymethyl	-0.75 [83]	5.3×10^{10} [84]	6.1×10^{9} (benzoquinone) [85]
		-0.90-1.18 [87]		$10^{4} – 10^{5}$ (formaldehyde) [85]
				1.75×10^{5} (H_2O_2) [88]
$C_4H_8OH^{\bullet}$	2-hydroxy-2-methylpropyl	0.09 [86]	-	2.2×10^{5} (H_2O_2) [88]
Fe^{3+}/Fe^{2+}	-	0.77 [89]	-	-
Fe^{4+}/Fe^{3+}	-	~3.4 [90]	-	-
Cu^{2+}/Cu^{+}	-	0.159 [89]	-	-

Cr^{3+}/Cr^{2+}	-	-0.42 [89]	-	-
$2Cr^{3+}/Cr_2O_7^{2-}$	-	-1,33 [89]	-	-

[1)] not bonded cations, only standard values

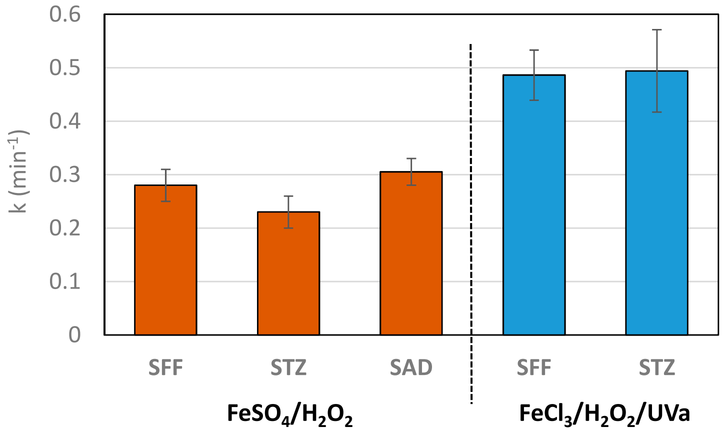

Figure S7. Comparison of the degradation rate constants od the selected SNs (0.1 mmol/l) during the Fenton process (0.15 mmol $FeSO_4$/l and 0.5 mmol H_2O_2/l) and Fenton-like reactions (photo Fenton, 1.0 mmol $FeCl_3$/l and 2.0 mmol H_2O_2/l)

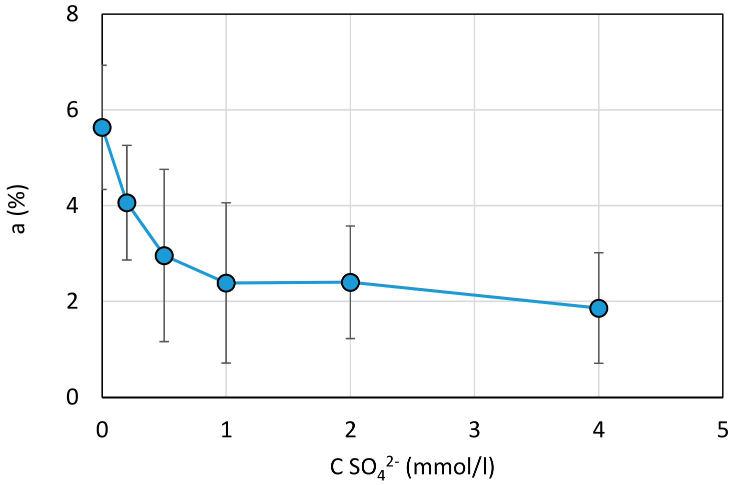

Figure S8. Effect of the SO_4^{2-} concentration on the sorption of SFF (0.1 mmol/l) on the catalytic system (0.5 g TiO_2/l /1.0 mmol $FeCl_3$/l) at pH 3.0±0.1

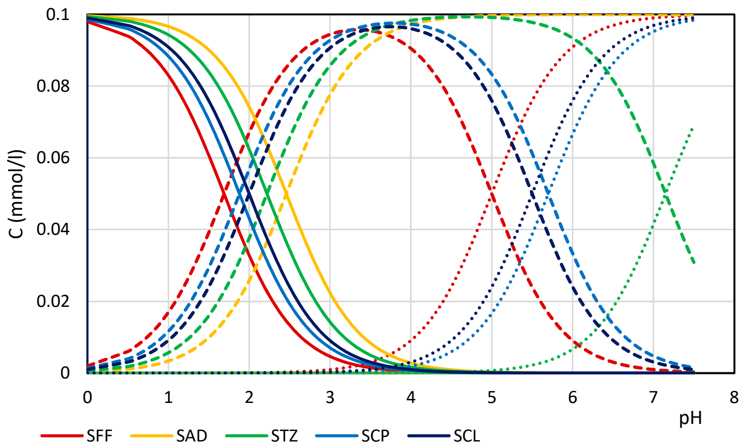

Figure S9. Effect of the pH on the dissociation and the concentration of molecular form of SNs (0.1 mmol/l) in aqueous solution: cationic form (SH_2^+) – solid line, neutral form (SH) – dashed line, anionic form (S^-) – dotted line

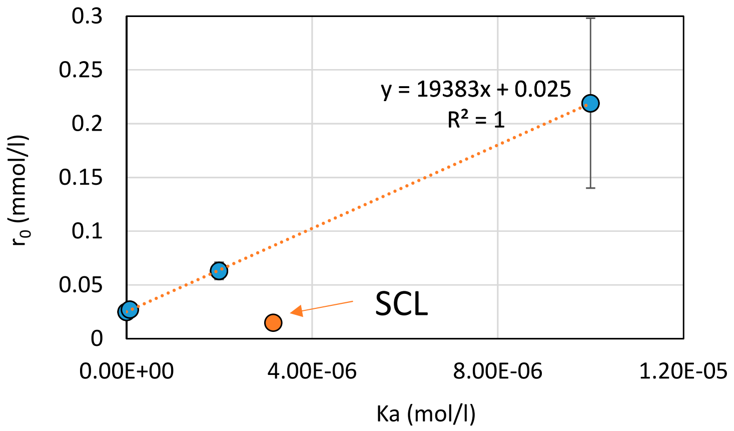

Figure S10. Relationship between the photodegradation rate of SNs (0.1 mmol/l) in the presence of a TiO_2(0.5 g/l)/$FeCl_3$(1.0 mmol/l) mixture and their dissociation constants (K_a)

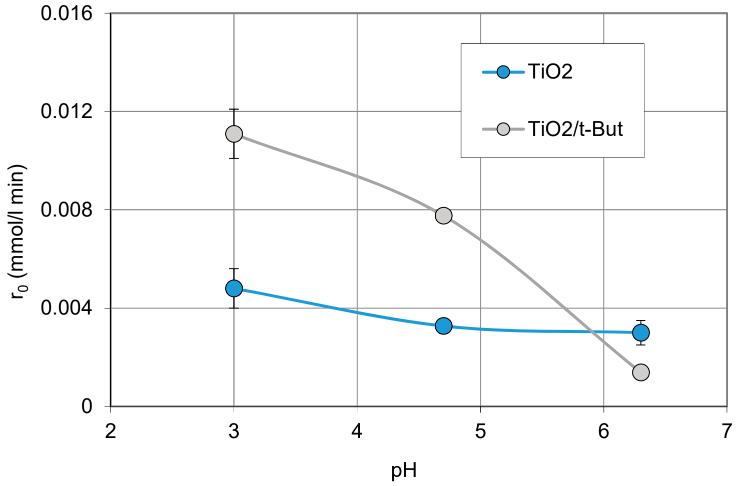

Figure S11. Effect of the pH on the initial rate of SFF photodegradation (0.1 mmol/l) in the presence of TiO_2 (0.5 g/l) or a TiO_2 (0.5 g/l)/t-But (10 mmol/l) mixture

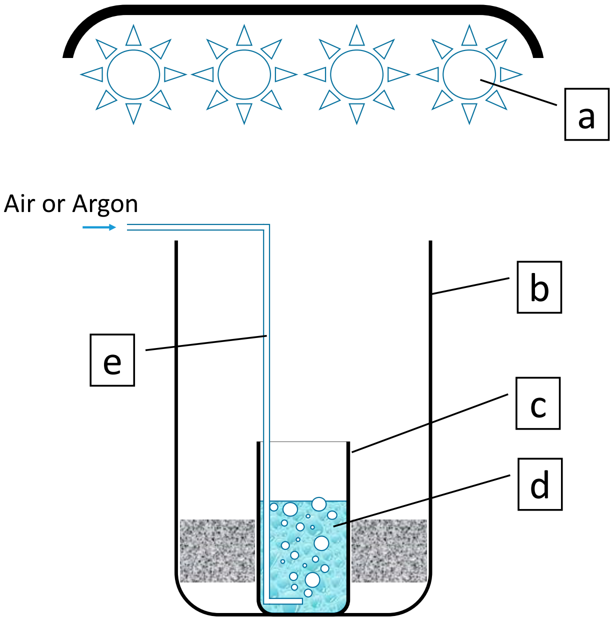

Figure S12. Test stand for the photocatalytic experiments under aerobic and anaerobic conditions, (a) UVa lamps; (b) a glass beaker (5000 ml); (c) a glass beaker (250 ml) (d) SN solution (150 ml, 0.1 mmol/l) with the selected catalyst system; (e) a glass tube (drain)

Table S5. Concentrations of substances used in studies of the photocatalytic degradation of SNs

Chemicals	Concentration (mmol/l)
tert-Butanol (t-But)	10
methanol	10
$CrCl_3{\cdot}6H_2O$	1.0
$AlCl_3{\cdot}6H_2O$	1.0
$CuCl_2{\cdot}2H_2O$	1.0
Na_2SO_4	3.0

Table S6. Detailed data for the UPLC procedure (gradient table).

	Time (min)	Flow rate (ml/min)	%A H_2O with 0.01% HCOOH	%B CH_3CN with 0.01% HCOOH
for SFF,SCL and SCP; ACQUITY UPLC BEH C18 column (130Å, 1.7 µm, 2.1 mm x 100 mm)				
1.	0	0.350	95.0	5.0
2.	3.5	0.350	80.0	20.0
3.	6.5	0.350	50.0	50.0
4.	7.5	0.350	50.0	50.0
5.	8.5	0.350	95.0	5.0
for SAD and STZ; ACQUITY UPLC BEH C18 column (130Å, 1.7 µm, 2.1 mm x 50 mm)				
1.	0	0.350	95.0	5.0
2.	3.0	0.350	60.0	40.0
3.	3.3	0.350	20.0	80.0
4.	3.5	0.350	95.0	5.0

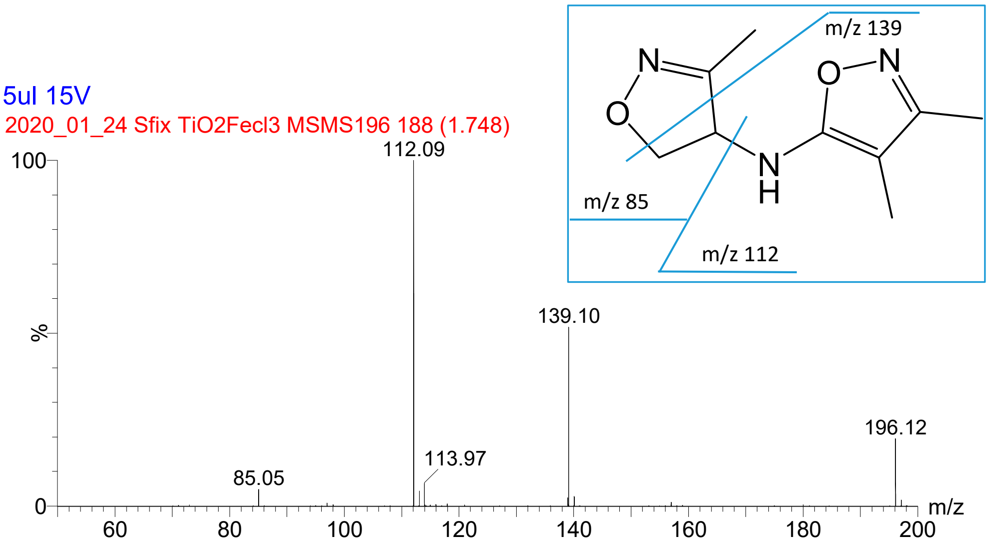

Figure S13. MSMS spectrum and a possible formula of compound with m/z 196.1086 Da occurred in SFF solution after irradiation for 90 s in the presence of a $TiO_2/FeCl_3$ mixture
